# A Lagrangian meshfree method applied to linear and nonlinear elasticity

**DOI:** 10.1371/journal.pone.0186345

**Published:** 2017-10-18

**Authors:** Wade A. Walker

**Affiliations:** Independent Researcher, Austin, Texas, United States of America; University of Michigan, UNITED STATES

## Abstract

The repeated replacement method (RRM) is a Lagrangian meshfree method which we have previously applied to the Euler equations for compressible fluid flow. In this paper we present new enhancements to RRM, and we apply the enhanced method to both linear and nonlinear elasticity. We compare the results of ten test problems to those of analytic solvers, to demonstrate that RRM can successfully simulate these elastic systems without many of the requirements of traditional numerical methods such as numerical derivatives, equation system solvers, or Riemann solvers. We also show the relationship between error and computational effort for RRM on these systems, and compare RRM to other methods to highlight its strengths and weaknesses. And to further explain the two elastic equations used in the paper, we demonstrate the mathematical procedure used to create Riemann and Sedov-Taylor solvers for them, and detail the numerical techniques needed to embody those solvers in code.

## Introduction

The repeated replacement method (RRM) [1] is a Lagrangian meshfree method for the simulation of time-dependent systems of conservation laws. We previously used RRM to simulate the one-dimensional Euler equations for compressible fluid flow to establish the basic functionality of the method, and we compared the results to an exact Riemann solver for the Euler equations given by Toro [2]. In this paper, we enhance RRM to increase its speed and accuracy, and apply it to the more challenging problems of one-dimensional linear and nonlinear elasticity. This allows us to demonstrate that RRM works for a range of constitutive equations, while maintaining good accuracy and error scaling behavior.

In this paper, we first motivate and derive our linear and nonlinear elastic constitutive equations, to define the terms and symbols used in subsequent sections. Second, we give anoverview of the repeated replacement method with a detailed comparison to earlier work, highlighting how the strengths and weaknesses of RRM differ from those of previous methods. Third, we explain the improvements made to RRM in the course of adapting it to handle elastic systems. Fourth, we present the derivation and explanation of our Riemann and Sedov-Taylor solvers. Then we show the results of RRM for eleven test problems, validating the results against Riemann and Sedov-Taylor solvers, and show that RRM’s error convergence behavior is somewhat super-linear. Finally, we present a summary and conclusion.

## Elastic equations

For both linear and nonlinear cases, we assume a homogeneous, one-dimensional, elastic continuum material that is subject to finite strain, which is a strain large enough that we cannot simplify our mathematical treatment by assuming it to be infinitesimal. The material’s reference (or undeformed) density is *ρ*_0_, and its spatial (or deformed) density at each point *x* is *ρ*(*x*). We will express the deformation in terms of the stretch λ, which is written in terms of density as λ(*x*) = *ρ*_0_/*ρ*(*x*). For a finite-sized piece of material, stretch is defined as λ ≡ *l*/*l*_0_, where *l*_0_ and *l* are the undeformed and deformed lengths of the piece, respectively. Strain is defined as *ϵ* ≡ (*l* − *l*_0_)/*l*_0_ = λ − 1.

We assume that the internal energy density of the material due to its state of strain and temperature, per unit of reference length, can be expressed as some function Ψ(λ, *T*) = Ψ_*s*_(λ) + Ψ_*t*_(λ, *T*), where Ψ_*s*_ is the strain energy density, and Ψ_*t*_ is the thermal energy density.

The Cauchy stress in such a material has both an elastic and a thermal component, and can be derived from the energy density as

$$\begin{array}{c} \sigma \left(\lambda ,T\right)=\sigma_{e}\left(\lambda \right)+\sigma_{t}\left(\lambda ,T\right)=\frac{\partial \Psi_{s}\left(\lambda \right)}{\partial \lambda }+\frac{\partial \Psi_{t}\left(\lambda ,T\right)}{\partial \lambda } \end{array}$$ (1)

A material whose stress is derived from an energy density function in this way is called a Green-elastic or hyperelastic material.

### Linear elasticity

Linear elasticity is defined by the direct proportionality of stress to strain. The elastic part of the stress is

$$\begin{array}{c} \sigma_{e}\left(\lambda \right)=E\epsilon =E\left(\lambda -1\right) \end{array}$$ (2)

where *E* is the elastic modulus, which has the units (*kg* ⋅ *m*)/*s*^2^ in one dimension. To obtain the strain energy density, we note that the integral of stress over distance is strain energy, and strain energy density with respect to the reference length is simply *strain energy*/*l*_0_. So, making use of the fact that λ = *l*/*l*_0_, the strain energy density is

$$\begin{array}{c} \Psi_{s}\left(\lambda \right)=\frac{1}{l_{0}}\int_{l_{0}}^{l}\sigma \left(l\right)\;\text{d}l=\frac{E}{2}{\left(\lambda -1\right)}^{2} \end{array}$$ (3)

To complete this simple model, we choose a thermal energy density Ψ_*t*_(*T*) = *C*_*v*_ *T* where *C*_*v*_ is the heat capacity in *J*/(*m* ⋅ *K*). This Ψ_*t*_ has no dependence on λ, since it is difficult to form such an expression that has the correct signs for both energy and stress equations without making the stress nonlinear in λ. This results in a total stress of

$$\begin{array}{c} \sigma \left(\lambda \right)=\frac{\partial \Psi_{s}\left(\lambda \right)}{\partial \lambda }+\frac{\partial \Psi_{t}\left(T\right)}{\partial \lambda }=E\left(\lambda -1\right) \end{array}$$ (4)

Note that this stress does not model thermal expansion, and also allows the material to be compressed down to zero length with finite work Ψ_*s*_(λ)|_λ = 0_ = *E*/2, so we have sacrificed physicality for the sake of linearity in this simple model.

The total energy density in spatial coordinates, including kinetic energy, is

$$\begin{array}{c} \Psi_{tot}\left(\lambda ,u,T\right)=\frac{\rho u^{2}}{2}+\frac{\Psi (\lambda ,T)}{\lambda }=\frac{\rho u^{2}}{2}+\frac{E}{2\lambda }{\left(\lambda -1\right)}^{2}+\frac{C_{v}T}{\lambda } \end{array}$$ (5)

where *u* is velocity, and the division of the Ψ term by λ is needed to convert the energy density from reference to spatial coordinates. Finally, making use of the fact that λ = *ρ*_0_/*ρ*, the speed of sound in the material in terms of the density is

$$\begin{array}{c} a\left(\rho \right)=\sqrt{\frac{\partial \sigma }{\partial \rho }}=\frac{\sqrt{E\rho_{0}}}{\rho } \end{array}$$ (6)

### Nonlinear elasticity

There are many existing models of nonlinear elasticity. Some, such as that of Ogden [3], are complex enough to form an energy density from any desired polynomial in λ to better match the behavior of real materials. In our case, we are merely trying to test a numerical scheme, not model a specific material, so instead of adopting a general model, we modify the linear elastic equation only as much as needed to prevent manifestly unphysical behavior. Specifically, if we change the −1 in the linear elastic stress to −1/λ_2_, we obtain

$$\begin{array}{c} \sigma_{e}\left(\lambda \right)=E\left(\lambda -\frac{1}{\lambda^{2}}\right) \end{array}$$ (7)

This new nonlinear stress approaches −∞ as the stretch approaches zero, which prevents the material from being compressed to zero length with a finite amount of energy. Integrating this stress the same way we did for linear elasticity, we obtain the strain energy density $\Psi_{s}\left(\lambda \right)=\frac{E}{2\lambda }{\left(\lambda -1\right)}^{2}\left(\lambda +2\right)$.

To model the fact that real materials expand when heated, we add a thermal energy density term Ψ_*t*_(λ, *T*) = *C*_*v*_ *T*/λ. The division by λ is because we want the sign of this term to become negative in the stress equation to reflect this expansion.

The total stress *σ* is again obtained from the energy density function by

$$\begin{array}{c} \sigma \left(\lambda ,T\right)=\frac{\partial \Psi_{s}\left(\lambda \right)}{\partial \lambda }+\frac{\partial \Psi_{t}\left(\lambda ,T\right)}{\partial \lambda }=E\left(\lambda -\frac{1}{\lambda^{2}}\right)-\frac{C_{v}T}{\lambda^{2}} \end{array}$$ (8)

and the total energy density in spatial coordinates is again given by

$$\begin{array}{c} \Psi_{tot}\left(\lambda ,u,T\right)=\frac{\rho u^{2}}{2}+\frac{\Psi (\lambda ,T)}{\lambda }=\frac{\rho u^{2}}{2}+\frac{E}{2\lambda^{2}}{\left(\lambda -1\right)}^{2}\left(\lambda +2\right)+\frac{C_{v}T}{\lambda^{2}} \end{array}$$ (9)

Finally, the speed of sound in the material in terms of the density and temperature is

$$\begin{array}{c} a\left(\rho ,T\right)=\sqrt{\frac{\partial \sigma }{\partial \rho }}=\sqrt{\frac{E\rho_{0}}{\rho^{2}}+\frac{2(E+C_{v}T)\rho }{\rho_{0}^{2}}} \end{array}$$ (10)

Note that the dependence of the thermal stress *σ*_*t*_ on λ is what causes the *T* term to appear in the speed of sound, without which the material would only support shock waves and not rarefactions. We demonstrate this in the course of deriving our Riemann solvers.

## Overview of the repeated replacement method

RRM is based on the observation that when we model conservation laws using a field of piecewise-constant cells, the primitive values in the cells’ interiors do not change directly. Instead, wavefronts of change expand out from the edges between adjacent, dissimilar cells, where the spatial derivative is nonzero. These expanding wavefronts carry primitive value changes into the cells’ interiors.

To motivate this statement, consider the following. As we will see in the section on the derivation of Riemann solvers, we can write a system of conservation laws in the form ***W***_*t*_ + ***A***(***W***)***W***_*x*_ = **0**, where ***W*** denotes a vector of primitive variables, ***A***(***W***) is a Jacobian matrix derived from the constitutive equations, and the subscripts represent partial differentiation with respect to *t* and *x*. From this equation, we can see that if the spatial derivative ***W***_*x*_ of the field is zero at a point, then the temporal derivative ***W***_*t*_ of the field must also be zero at that point. Since our cells are piecewise constant, the spatial derivative of the field is zero everywhere except at the edges between cells, so time evolution can only begin at these edges.

In RRM, we track the expansion of these wavefronts over time. Once a wavefront reaches a certain width, we chop out the cells and parts of cells it encompasses, and replace them with a new piecewise-constant cell containing the same mass, momentum, and energy. The more different the adjacent cells are, and the lower the user has set the maximum allowable error, the less a wavefront is allowed to expand before it is replaced by a new cell. Each new cell gives rise to two new wavefronts, one at each edge.

To process these wavefronts, RRM uses an event-based simulator. Wavefronts are stored in a queue, ordered by the wavefronts’ replacement time. Each simulation event consists of updating the simulation time to the replacement time of the soonest wavefront in the queue, removing that wavefront from the queue, using it to create a new cell in the field, and adding the new cell’s two new wavefronts back into the queue. Continuing this process drives the time evolution of the field. The following sections describe this process in greater detail.

### Cells, wavefronts and tracer particles

**Fig 1:**
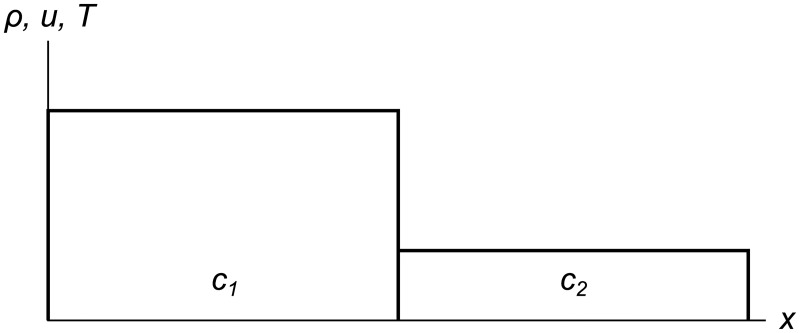
Two-cell initial conditions. Domain subdivided into two constant-valued cells *c*_1_ and *c*_2_. The *ρ*, *u*, and *T* variables are all shown on one axis for brevity, even though in general they have different values.

RRM subdivides the domain into cells *c*_*i*_, each of which contains constant values of the primitive variables ***W*** = [*ρ* *u* *T*]^*T*^. A simple two-cell set of initial conditions is shown in Fig 1. At the start of the simulation, the cells are typically chosen to have no gaps or overlaps between them. But since this is a Lagrangian meshfree method, the cells are what moves, not a mesh, so gaps and overlaps between cells will appear and disappear over the course of the simulation.

**Fig 2:**
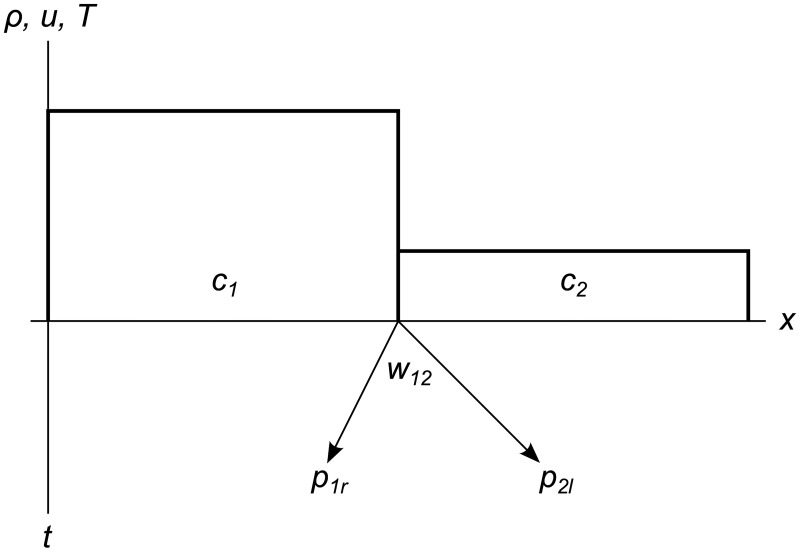
Expanding wavefront. Wavefront *w*_12_ and tracer particles *p*_1*r*_ and *p*_2*l*_ expanding from the edges between cells *c*_1_ and *c*_2_. Tracer particle *p*_1*r*_ moves at speed *a*(***W***_1_), and tracer particle *p*_2*l*_ moves at speed *a*(***W***_2_). The left and right bounds of the domain are inactive in this example, otherwise we would show wavefronts expanding from them as well.

If we write a system of conservation laws as ***W***_*t*_ + ***A***(***W***)***W***_*x*_ = **0**, we can see that $\frac{\partial W}{\partial t}=0$ if $\frac{\partial W}{\partial x}=0$, so no evolution takes place inside constant-valued cells. Instead, all evolution originates at the cell edges where the spatial derivative is nonzero. For the edges between *c*_1_ and *c*_2_, we trace out an expanding wavefront *w*_12_ using a pair of tracer particles *p*_1*r*_ and *p*_2*l*_ moving at the speed of sound in *c*_1_ and *c*_2_, respectively, as shown in Fig 2. We call these “tracer” particles because they are just an aid to simulation, rather than a representation of real physical particles.

For the tracer particles, we define an error metric

$$\begin{array}{c} \Delta_{1,n}=\sum_{i=1}^{n}d_{i}\left|W_{i}-W_{i-1}\right| \end{array}$$ (11)

which gives a measure of the distance-weighted total variation in primitive values which the tracer particle encounters as it moves from cell *c*_1_ to cell *c*_*n*_, where cell *c*_0_ is the cell on the opposite side of the wavefront where the tracer particle was created, *d*_*i*_ is the distance traveled by the tracer particle inside cell *c*_*i*_, and ***W***_*i*_ is the vector of primitive values for cell *c*_*i*_.

**Fig 3:**
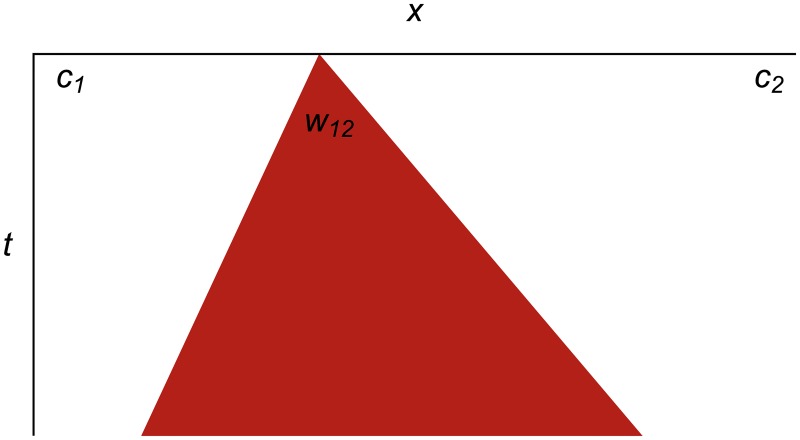
Expanding wavefront as a spacetime diagram. Spacetime diagram showing wavefront *w*_12_ expanding from the edges between cells *c*_1_ and *c*_2_, which are represented implicitly as white areas. The tracer particles travel down the edges of the colored wavefront triangles, but are not explicitly shown on these diagrams.

Fig 3 shows what that same wavefront expansion looks like as a spacetime diagram. In this format, we make the cells implicit as white areas, and show wavefronts as colored triangles expanding in space as the time increases going downward. This format will allow us to represent many hundreds of events succinctly on one diagram.

**Fig 4:**
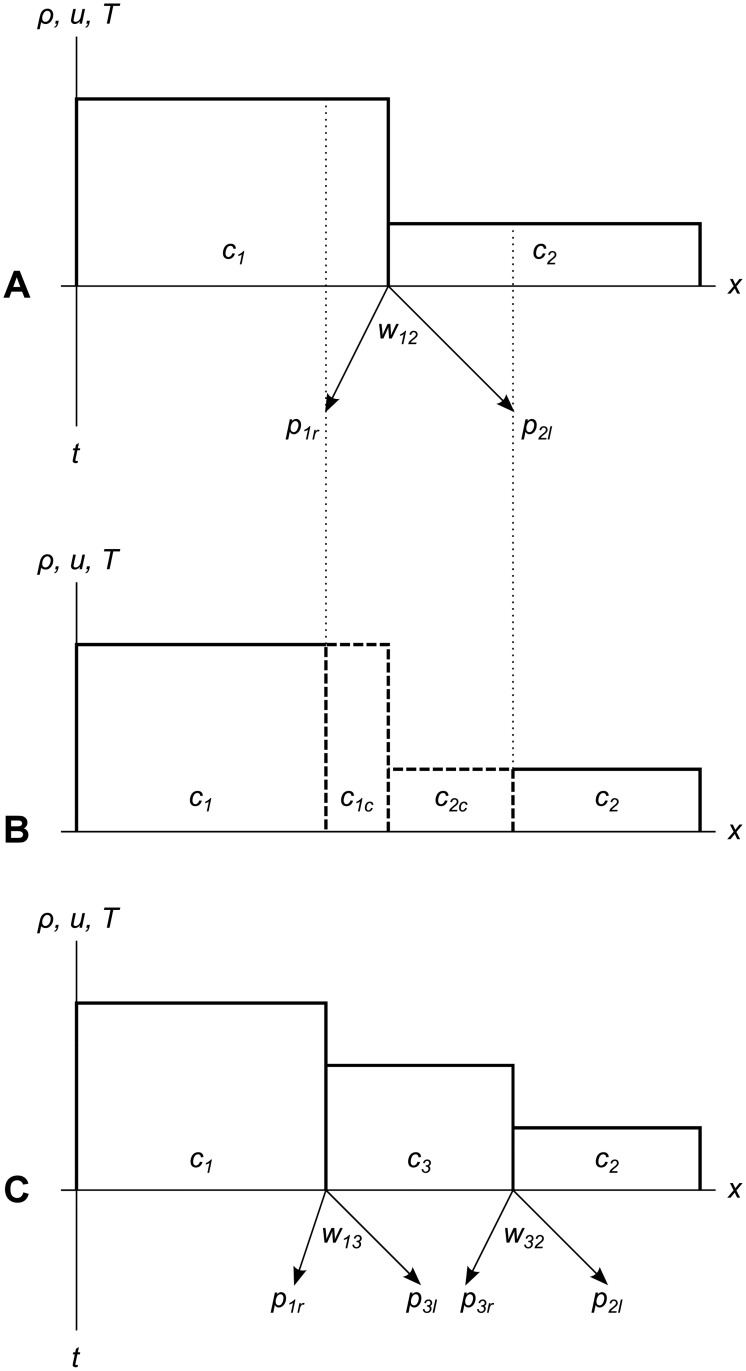
Wavefront chopping and flattening. A wavefront chopping off parts of two cells to replace them with a new cell. (A) Wavefront *w*_12_ sends tracer particles *p*_1*r*_ and *p*_2*l*_ out into cells *c*_1_ and *c*_2_. (B) Wavefront *w*_12_ reaches its error metric limit **Δ**_*max*_ and chops piece *c*_1*c*_ off of cell *c*_1_, and piece *c*_2*c*_ off of cell *c*_2_. (C) Conserved quantities from pieces *c*_1*c*_ and *c*_2*c*_ are added up and flattened to form new cell *c*_3_. The new cell has new wavefronts *w*_13_ and *w*_32_ at its left and right edges, respectively. Each new wavefront has two new tracer particles.

**Fig 5:**
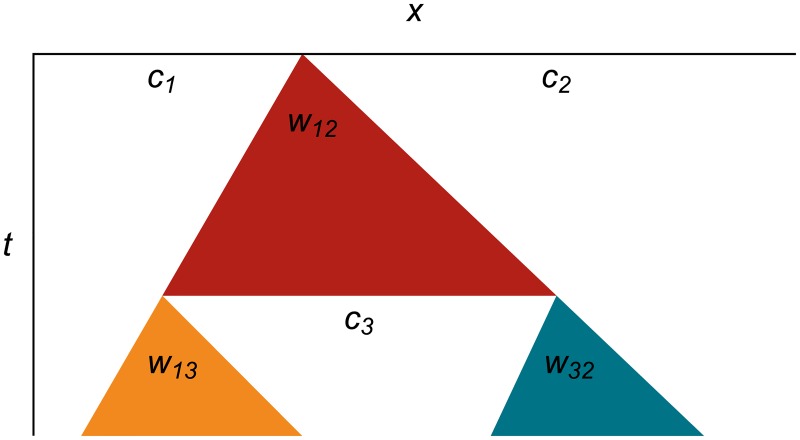
Wavefront expansion, chopping and replacement as a spacetime diagram. Wavefront *w*_12_ expands until it reaches its error metric limit, then chops pieces off of cells *c*_1_ and *c*_2_ and replaces them with a new cell *c*_3_. The new cell has new wavefronts *w*_13_ and *w*_32_ at its left and right edges, respectively.

Once a wavefront gets so wide that the error metric of one of its tracer particles exceeds some user-defined limit **Δ**_*max*_, we chop out the cells and parts of cells under the wavefront, accumulate all their conserved quantities, and replace that area with a new constant-valued cell. This “flattening” process is shown in Fig 4. If our simulation includes multiple material types, the flattening process may produce multiple new constant-valued cells, one for each connected area of a single material type. The same flattening process is shown as a spacetime diagram in Fig 5.

Conversion between momentum, kinetic energy, and potential energy is modeled using the stress momentum *P*_*σ*_ = *σ*Δ*t* and stress energy *E*_*σ*_ = *σu*Δ*t*, which we store in each cell in addition to the primitive values. Each cell *c*_*i*_ initially contains $P_{\sigma c_{i}l}=\sigma_{i}\frac{w_{i}}{a_{i}}$ and $E_{\sigma c_{i}l}=\sigma_{i}u_{i}\frac{w_{i}}{a_{i}}$ directed to the left, and $P_{\sigma c_{i}r}=-\sigma_{i}\frac{w_{i}}{a_{i}}$ and $E_{\sigma c_{i}r}=-\sigma_{i}u_{i}\frac{w_{i}}{a_{i}}$ directed to the right, where *w*_*i*_ is the width of the cell and *a*_*i*_ = *a*(***W***_*i*_) is the speed of sound in the cell. These quantities sum to zero for each cell, so they do not affect the overall amount of momentum and energy in the simulation. Tracking *P*_*σ*_ and *E*_*σ*_ in this way allows conservation of momentum and energy to be checked at every simulation event, even though the entire field is never updated simultaneously. Note that *P*_*σ*_ and *E*_*σ*_ are not precisely fluxes, since they do not cross cell boundaries, but they do serve a similar purpose in that they drive the time evolution of the field.

During flattening, each cell edge inside the wavefront contributes *P*_*σ*_ = ±*σ*Δ*t* and *E*_*σ*_ = ±*σu*Δ*t* to the flattening process, where Δ*t* is the amount of time since the cell was created, or since that side of the cell was last chopped. The total mass, momentum and energy *M*, *P*, *E* accumulated by one wavefront chop of cells *c*_1_ through *c*_*n*_ by wavefront *w*_1*n*_ are

$$\begin{array}{c} \begin{array}{cc} & M=M_{c_{1c}}+M_{c_{2}}+\ldots +M_{c_{n-1}}+M_{c_{nc}}\\ & P=P_{c_{1c}}+P_{\sigma c_{1}r}+P_{\sigma c_{2}l}+P_{c_{2}}+\ldots +P_{c_{n-1}}+P_{\sigma_{n-1}r}+P_{\sigma c_{n}l}+P_{c_{nc}}\\ & E=E_{c_{1c}}+E_{\sigma c_{1}r}+E_{\sigma c_{2}l}+E_{c_{2}}+\ldots +E_{c_{n-1}}+E_{\sigma_{n-1}r}+E_{\sigma c_{n}l}+E_{c_{nc}} \end{array} \end{array}$$ (12)

where *M*_*c*_1*c*__ and *M*_*c*_*nc*__ are the partial masses chopped from *c*_1_ and *c*_*n*_, respectively, by the wavefront, and *M*_*c*_2__ through *M*_*c*_*n* − 1__ are the entire masses of cells *c*_2_ through *c*_*n* − 1_ which are subsumed by the wavefront. A similar notation holds for the momenta *P* and energies *E*, using the energy density Ψ_*tot*_ from the elastic model for the chopped energy. So for example *P*_*σc*_1_*r*_ + *P*_*σc*_2_*l*_ is the net stress momentum across the edge between cells *c*_1_ and *c*_2_, and *E*_*σc*_1_*r*_ + *E*_*σc*_2_*l*_ is the net stress energy across the edge between cells *c*_1_ and *c*_2_.

Once we have accumulated the conserved quantities, we can determine the primitive values of the new cell algebraically by

$$\begin{array}{c} \begin{array}{cccccc} \rho & = & \frac{M}{w_{w}} & u & = & \frac{P}{M}\\ KE & = & \frac{1}{2}Mu^{2} & PE & = & E-KE\\ \lambda & = & \frac{\rho_{0}}{\rho } & l_{0} & = & \frac{w_{w}}{\lambda }\\ T & = & \left(\frac{PE}{l_{0}}-\Psi_{s}\right)\left(\frac{1}{\Psi_{t}{|}_{T=1}}\right) \end{array} \end{array}$$ (13)

where *w*_*w*_ is the width of the wavefront at replacement time, *KE* and *PE* are the kinetic and potential energies of the new cell, respectively, and Ψ_*s*_ and Ψ_*t*_ are taken from the appropriate elastic model.

In the case where more than one type of material is present inside a wavefront during flattening, we create a new cell for each contiguous area of each material type, and flatten those areas separately, with the exception of stress momentum and stress energy, which are divided among the new cells in proportion to their size.

### Early replacement and wavefront merging

When two adjacent cells have very different primitive values, the wavefront created across their shared edges will be replaced quickly, resulting in the creation of a relatively small new cell and an overall increase in the number of cells in the simulation. This is called early replacement, and is the case shown in Fig 4.

**Fig 6:**
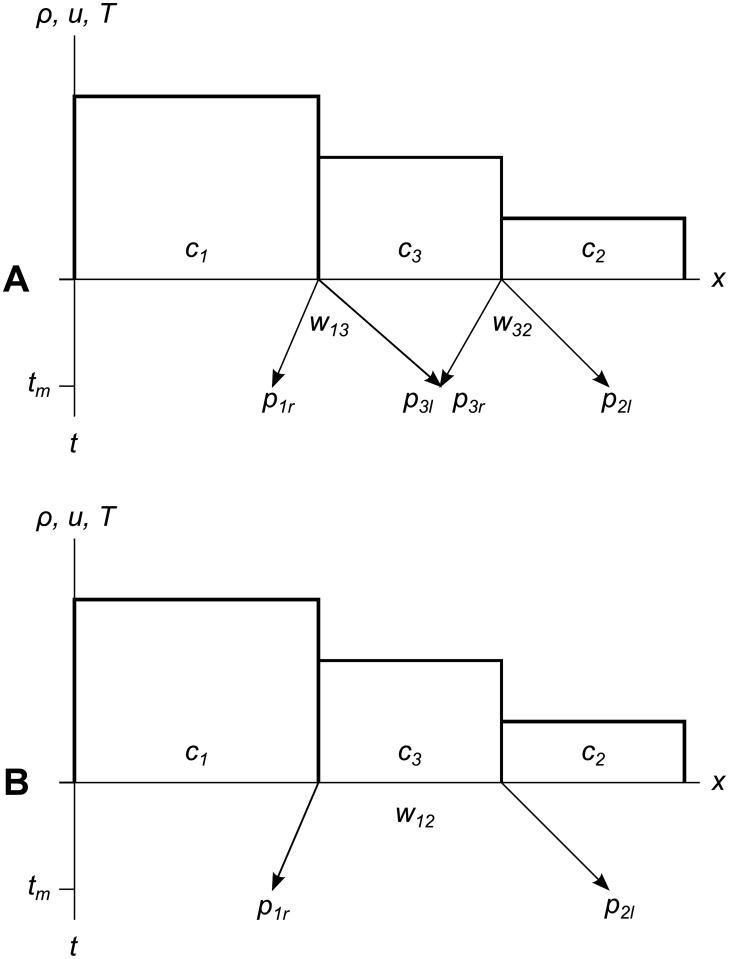
Two wavefronts merging to form one larger wavefront. (A) Wavefronts *w*_13_ and *w*_32_ intersect at time *t*_*m*_, before any of the tracer particles reaches the error metric limit **Δ**_*max*_. (B) No chopping is required yet, but we do not want the wavefronts to overlap, because since only the outer particles would define the edges of the eventual new cell, tracking the inner particles would be redundant. So we create a new wavefront *w*_12_ by merging wavefronts *w*_13_ and *w*_32_, which removes the inner particles *p*_3*l*_ and *p*_3*r*_.

In areas where cells’ primitive values are similar, adjacent wavefronts will intersect before the error metric grows large enough to require replacement. To reduce the number of tracer particles we must track during simulation, we do not allow wavefronts to overlap, so we merge intersecting wavefronts together unless their summed error metrics would exceed the user-defined limit **Δ**_*max*_. This wavefront merging results in a decrease in the number of particles and cells in the simulation, and is illustrated in Fig 6. Any number of wavefronts may be merged together in this way, so long as the total error metric remains lower than **Δ**_*max*_.

**Fig 7:**
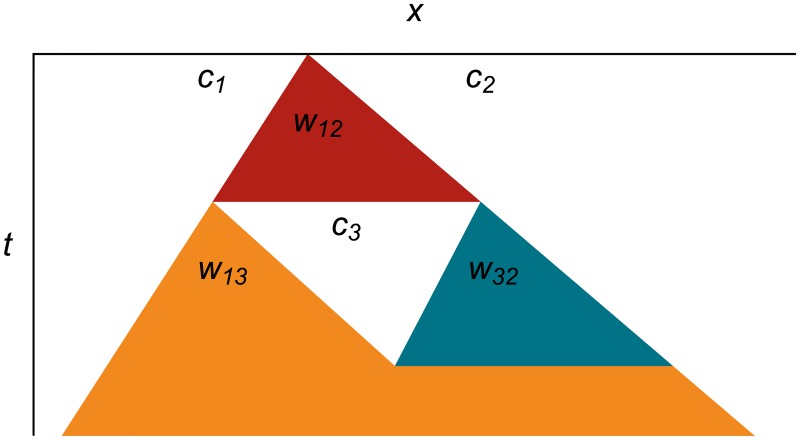
Wavefront life cycle as a spacetime diagram. Wavefront *w*_12_ expands into cells *c*_1_ and *c*_2_, then chops and flattens parts of them to form cell *c*_3_, which spawns two new wavefronts *w*_13_ and *w*_32_ at its left and right edges, respectively. The new wavefronts expand until they completely cover *c*_3_, then merge to leave only one wavefront, which continues to expand.

Fig 7 shows an entire cycle of wavefront expansion, flattening, new wavefront creation, and wavefront merging in one spacetime diagram. We will use this more succinct format when we show long sequences of events.

The interaction between early replacement and wavefront merging is what makes RRM adapt to local conditions across the field. And since replacement and merging are scheduled by event-based simulation instead of a global time tick, greater activity in one area need not affect other areas. The current implementation of RRM uses a single event queue, but events are only dependent if their wavefronts are spatially adjacent, so this event queue could be broken up into separate queues for different regions of the field to parallelize the algorithm.

An intentional result of wavefront merging is that it allows RRM to preserve a constant-valued field exactly. In other words, it allows RRM to satisfy the geometric conservation law (GCL), so named by Thomas and Lombard [4] and more recently discussed by Guillard and Farhat [5]. We can see that in a constant-valued field, stress momentum *P*_*σ*_ and stress energy *E*_*σ*_ across any pair of cell edges will sum to zero, since the cells’ primitive values are equal. So then Eqs (12) and (13) tell us that if we chop out any wavefront in a constant-valued field, it will encompass an amount of material which, upon flattening, will yield a new cell with the same primitive values. However, this is only true if wavefronts are not allowed to overlap. If they do overlap, it is possible for a wavefront in a constant-valued field to encompass unbalanced stress momentum and stress energy, unless all the overlapping wavefronts are unioned and replaced as one, as mentioned in the section on improvements to RRM.

### Positivity preservation

We say that a scheme is positivity-preserving if it is guaranteed never to produce a negative value for a quantity which should not be negative, such as density, pressure, or temperature. For density, RRM is always positivity-preserving, since new cells’ masses are added up from chopped parts of other cells. And for the Euler equations, though we occasionally see a negative pressure upon flattening, those negative pressures are the result of numerical error and are on the order of the machine precision. But for nonlinear elasticity, negative temperatures can occur because of the form of the energy density Eq (9), which is dependent upon both stretch λ and temperature *T*. In the Euler equations, once a cell’s size, mass, and velocity have been determined, any remaining energy can be assigned to potential energy simply by setting the pressure *p* to the necessary value. But in nonlinear elasticity, for a specific cell stretch and mass, a specific amount of strain potential energy is required, which may not leave enough energy for the thermal potential energy at that same stretch. The result is a negative temperature.

A positivity analysis shows that this effect is most pronounced at low temperatures, when neighboring cells are very different in density and velocity. Interestingly, the effect does not depend on how long a wavefront has been allowed to expand, so it cannot be remedied simply by increasing the temporal precision. Instead, the effect seems to be inherent in the constitutive equation itself, which may point to an additional constraint we should impose during the construction of such equations.

In the test cases we examined, negative temperatures only occur near contacts of dissimilar materials, since the lack of heat diffusion across the contact restricts the energy available to each flattened cell. The solution is to add a small amount of thermal energy to bring the flattened cell up to *T* = 0, and then to maintain energy conservation by adding a corresponding negative strain energy to the flattened cell. This negative energy is carried forward a short time into the future, until sufficient energy is encompassed by nearby wavefronts to cancel it out. We use the same solution for the smaller numerical errors.

### Flowchart

**Fig 8:**
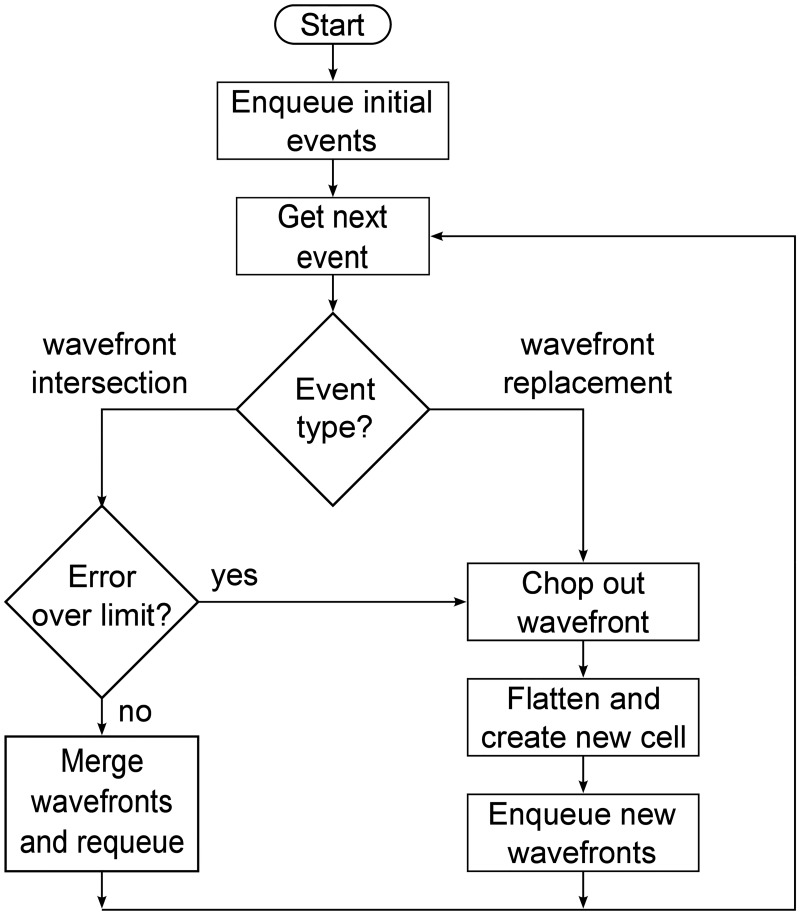
Event flowchart. Summarizes the processing of events during simulation.

Fig 8 shows the processing of events during simulation. The initial events are those enqueued for the wavefronts spanning the edges of the initial condition cells. The event queue is arranged in order of increasing wavefront replacement time, so the soonest event is always the next one in the queue, though new events may be inserted at any position.

To keep the flowchart readable, we omit a few optimizations that are present in the current implementation of RRM. For example, if two wavefronts intersect, but their combined error would be over the limit, they are chopped out into two adjacent new cells instead of being merged. And at the edge between the two new cells, only one new wavefronts is created instead of two, to avoid two identical new wavefronts on top of each other.

### Comparison to previous work

RRM was designed for use on systems of conservation laws that are mathematically inconvenient, so it requires only that the system have conserved quantities that can be numerically integrated, and that some expression for the speed of sound can be obtained. RRM does not require the evaluation of a Riemann solver, so it may be applied to conservation equations for which the Riemann solver derivation procedure is intractable. RRM also does not require the evaluation of spatial or temporal derivatives, so it may be applied to equations where such derivatives are expensive or not well defined at every point.

RRM was also designed to be adaptive. Temporally, it is unconditionally stable for time steps of any length, though it loses accuracy as the time step length is increased. Spatially, it does not require a mesh, and it can add or remove cells wherever they are needed to maintain accuracy, without the requirement to stitch together or nest meshes of different resolutions.

Of course, advantages generally come paired with disadvantages. RRM’s main disadvantages are its high computational intensity, high programming complexity, and low-order reconstruction of solutions.

The largest difference between RRM and methods such as the finite difference (FD) method, finite volume (FV) method, and the finite element method (FEM) is that RRM does not assemble a system of explicit or implicit simultaneous equations for some area of the field and then apply a solver to find the vector of unknown primitive values for that whole area at each time step. Instead, RRM is a purely local method, where wavefronts are added and removed only as required to meet the user’s accuracy goals, and the temporal and spatial steps can be different for every wavefront.

Another major difference is that RRM does not explicitly bound its time steps using the Courant-Friedrichs-Lewy (CFL) condition, and such a condition is not required for stability. RRM does track the intersection of wavefronts, but if the intersecting wavefronts are sufficiently similar, they may simply be merged together, which causes a loss of accuracy, but does not affect stability. Since wavefronts travel at the speed of sound, their intersection generally happens at a time later than the “signal time” used in codes like Whitehurst’s FLAME [6], which is based on the time taken for opposing shocks to traverse a cell and intersect within it. RRM cannot use the signal time because it does not solve the Riemann problem across the cell edges, so it does not know the shock speeds. However, RRM does track the motion of cell edges, and in cases where two cells interpenetrate at a speed faster than the speed of sound, the wavefront emanating from that pair of edges is broadened so that it always encompasses them.

RRM is probably most similar to cell-centered Lagrangian finite volume schemes such as those described by Godunov [7], then later by Després and Mazeran [8], Maire et al. [9], Maire [10], Carré et al. [11], Kluth and Després [12], Burton et al. [13] [14], and Vilar et al. [15], among others. The main difference between these schemes and RRM is that instead of changing the primitive values and shapes of existing cells based on momentum and energy fluxes across the cell faces, in RRM cells are unchanged after their creation, except for having parts chopped off to form new cells. In RRM the stress momentum *P*_*σ*_ and stress energy *E*_*σ*_ play a role similar to momentum and energy flux in Lagrangian finite volume schemes, but strictly speaking they are not fluxes, since they do not cross from one existing cell to another. Instead, they are one source of the conserved quantities used to form new cells, the other source being the chopped-off parts of other cells.

As a Lagrangian method, RRM automatically achieves Galilean invariance, whereas in some Eulerian methods the bulk velocity of the material may alter the simulation results, as noted by Wadsley et al. [16]. And unlike other adaptive methods such as adaptive mesh refinement (AMR) [17], RRM does not require cell sizes or time steps at different refinement levels to be multiples of each other, and does not require grids of varying degrees of refinement to be nested in any specific way.

Some variants of the finite element method such as those of Peraire et al. [18] and Radovitzky and Ortiz [19] support fully Lagrangian operation. These methods use adaptive and frequent remeshing to prevent the mesh from becoming tangled in areas of high deformation. The remeshing methods used, such as the advancing front algorithm, are paired with refinement indicators that attempt to evenly distribute quantities such as deformation among the elements of the mesh. This permits simultaneous coarsening and refinement of the mesh in different locations. However, remeshing the entire field is an expensive operation which must be carefully designed to minimize impact on simulation speed, and the transfer of conserved quantities from the old to the new mesh can introduce numerical diffusion. Such diffusion may be minimized by arbitrary Lagrangian-Eulerian (ALE) methods (originated by Hirt et al. [20] and recently reviewed by Barlow et al. [21]), which allow the mesh to move independently of the material only where required to fix mesh tangling.

Moving-mesh codes which are based on Delaunay or Voronoi tessellations of a set of moving points (such as FLAME [6], Springel’s AREPO [22], Duffell and MacFadyen’s TESS [23], and that of Gaburov et al. [24]) are similar to RRM in that a set of freely-moving objects serves as the basis of the field. However, these tessellation-based methods may require some care to insure that the point positions do not imply a degenerate mesh, and they may need to add points, fuse points, or steer the trajectories of the points to keep them near the centers of mass of their cells, thereby keeping the cells rounder and more tractable. Since RRM does not compute any derivatives, it does not require any constraints on cell motion or position to avoid degeneracy. However, RRM does need to prevent cells from being subdivided down to a size too close to machine precision, to insure that the edges of the cells remain distinguishable from each other when subjected to numerical error.

RRM is similar to AREPO [22] in that it can incrementally add or remove cells in specific areas of the field. In AREPO, these processes are called refinement (when a cell is split in two) and de-refinement (where a cell is removed and its conserved quantities distributed among its neighbors). The difference is that in RRM, cells are never enlarged, they are either nibbled away by neighbors over multiple simulation events, or are removed all at once after their left and right wavefronts intersect inside the cell.

RRM ensures the “manifestness” of conserved quantities by storing two extra quantities in each cell, the stress momentum *P*_*σ*_ and the stress energy *E*_*σ*_. As noted in the section on wavefronts and particles, these quantities insure that after each event, global momentum and energy are still exactly conserved, even if, for example, the event introduces some change in momentum or energy which has not yet been balanced by a corresponding event elsewhere in the field. Other codes such as AREPO [22] and Hopkins’ GIZMO [25] solve the same problem by requiring time steps to conform to a power-of-two hierarchy so that time steps at a given level of the hierarchy may be synchronized without synchronizing the entire field, and by requiring fluxes on both sides of a face to be updated in the same time step.

RRM is similar to discontinuous Galerkin (DG) methods [26] in that there is no global enforcement of continuity across cell boundaries as there is in the finite element method. Discontinuous Galerkin methods do assemble a matrix of equations which must be solved, but this matrix is typically block diagonal, so it may often be solved more simply than in the case of FEM.

RRM is somewhat similar to the original Eulerian version of the Godunov method [27], in that they both represent the field as piecewise-constant values, and both explicitly model time evolution at cell edges. RRM is simpler in one way, because instead of solving an exact or approximate Riemann problem at each set of edges, it only models a wavefront of two characteristics expanding outward at the speed of sound. For shock waves, Godunov’s method directly models the shock speed by recovering it from the underlying Riemann solver. As we will see in the results for a nonlinear shock tube, RRM achieves the correct shock speed as a dynamic phenomenon whereby a thin velocity and stress spike forms at the shock, but without explicitly calculating the shock speed. RRM is also Lagrangian instead of Eulerian, which makes it more complex to code than the original Godunov method since cells can move and overlap.

Compared to Riemann-solver-based high-order reconstruction schemes such as MPWENO (as used for nonlinear elasticity for example by Barton et al. [28]), RRM does not require slope limiters, multiple weighted stencils, or other special care to avoid oscillation near steep gradients, since its solution reconstruction is currently only piecewise constant. RRM can also be used in cases where solution of the Riemann problem is prohibitive, either mathematically or computationally. However RRM does suffer from a one-way overshoot at shocks, as we will see in the results for a nonlinear shock tube, so it could be argued that RRM could also benefit from some special treatment to avoid creating new extrema of the solution in that case. In principle, the cells of RRM could be made higher-order in a way similar to finite volume schemes, but we have not yet investigated this possibility.

RRM bears many similarities to other meshfree methods such as smoothed-particle hydrodynamics (SPH) [29] [30] and more recent kernel-based methods such as those of Lanson and Vila [31], Gaburov and Nitadori [32], and Hopkins [25]. The cells of RRM could be compared to the particles of SPH, with the wavefronts of RRM playing a role similar to SPH’s kernel functions. In SPH, for a locally-supported kernel function, the value of the field at a given point is determined by the weighted contributions of some number of nearby particles. In RRM, the “support” of a wavefront is the set of cells and parts of cells that it encompasses, but wavefronts never overlap, and the primitive values within a given wavefront depend only on that wavefront. RRM’s maintenance of connections between wavefronts and cells is similar to certain acceleration techniques used in SPH implementations to reduce the number of particles that must be examined to produce the field values at each point.

The Finite Mass Method (FMM) published by Gauger et al. [33] and Klingler et al. [34] is a meshfree method that uses extended pieces of material in a way somewhat similar to the cells of RRM. FMM divides the material being simulated into finite-sized “mass packets”, which are modeled by differentiable functions with compact support such as cubic B-splines. FMM packets can interpenetrate, as cells can in RRM, but FMM packets can also deform and change in size, whereas in RRM the cells are of fixed size and are chosen to be piecewise-constant specifically so their shape does not change over time. In FMM, packets are coupled by frictional forces, where in RRM the cells are independent of each other. The solution procedure of FMM is similar to that of FEM, where we assemble a system of differential equations containing the packet locations, deformation matrices, and entropies, and solve the system with standard techniques, as opposed to the event-based simulation of RRM. Finally, since FMM packets can change shape, they can become degenerate, in which case they must be recreated and the simulation continued, as noted by Klingler et al. [35].

RRM was partly inspired by work from computational geometry, specifically Chaikin’s corner-cutting algorithm [36] and Catmull’s subdivision surfaces [37]. Both algorithms start with simple curves and progressively refine them by performing some repeated, local operations. RRM adds the notion of conserving mass, momentum, and energy during the process, and can de-refine as well as refine, but shares a similar basic idea.

## Improvements to the repeated replacement method

There are several differences between the original version of RRM and the one described in this paper. Originally, RRM was demonstrated only for the Euler equations. In the process of adapting RRM to handle elasticity, we made a number of improvements.

The change that improved accuracy the most was the addition of support for multi-material fields. In its original form, RRM is diffusive across contacts, since the chopping and replacement process mixes together material which was initially at different temperatures. An example of this behavior can be seen in the double rarefaction test in the results section. Arguably this is physically accurate, since it models heat diffusion, but it is at odds with the analytic solutions we obtain from Riemann solvers, which are adiabatic. So to make RRM adiabatic, we added the option to mark cells as being of different material types. For initial conditions with two different temperatures, if we use a different material type for each temperature, it allows RRM to avoid mixing them together. This requires cell flattening to create as many new cells as there are contiguous areas of each material type inside the wavefront. So flattening across a contact will result in two adjacent cells, which keeps contacts sharp and removes the major source of error versus a Riemann solver. Most of the other tests in the results section make use of this feature. The error analysis section contrasts the error behavior with and without this feature to quantify the improvement it brings.

Another change from the original version of RRM is in the improved handling of wavefront intersection. Originally, tracer particles were allowed to cross each other and move from one cell to another. Then at replacement time, all the wavefronts that overlapped the replacing wavefront were unioned together and replaced as one. This unioning was needed to prevent wavefronts from sometimes chopping out an unbalanced amount of stress momentum and energy, which could cause large negative temperatures or violations of the geometric conservation law, as mentioned in the sections on wavefront merging and positivity preservation. But such unioning presented problems for shock-only systems like linear elasticity, where indiscriminate unioning of wavefronts could destroy the shocks. It was also difficult to implement particle motion between cells properly for cases where multiple cells overlapped or interpenetrated at high speeds. So we changed to a system where wavefronts never overlap, and must be explicitly merged together when they intersect. This gives us the option of wavefront replacement before merge to preserve a shock or to keep error below the user threshold. It also removes the requirement to track particle motion between cells or to support more than two particles in a single cell.

The change that improved the speed of RRM the most was the reduction of the computational order of the wavefront-cell intersection operation. The previous implementation of RRM was $O(N^{2})$ for this operation, because at replacement time it would intersect the wavefront with every other wavefront to check for overlaps, and with every cell to determine which cells were being removed from the field. The current implementation is O(N), because a replacing wavefront never overlaps another, and because we keep track of which cells each wavefront has crossed so we can remove them from the field without intersecting wavefronts against cells.

To demonstrate the speed improvement due to this change, we profiled the performance of the same test problem for the old and new versions of the simulator. Since the old simulator only supported the Euler equations, we chose a shock-tube problem with initial conditions (*ρ*_*l*_, *u*_*l*_, *p*_*l*_) = (1.0, 0.0, 100000.0), (*ρ*_*r*_, *u*_*r*_, *p*_*r*_) = (0.01, 0.0, 1000.0), with the ratio of specific heats set to *γ* = 1.4. The simulation domain is *x* ∈ [−1.0, 1.0], and the simulation time runs from *t* = 0.0 to *t* = 1.5 seconds. The precision settings were chosen such that the number of cells would exceed 1000, and were set the same in both versions of the simulator. The solution consists of a left rarefaction, a contact, and a right shock. Both tests were run on a single core of an Intel Core i7-3820 running at 3.6 GHz.

**Fig 9:**
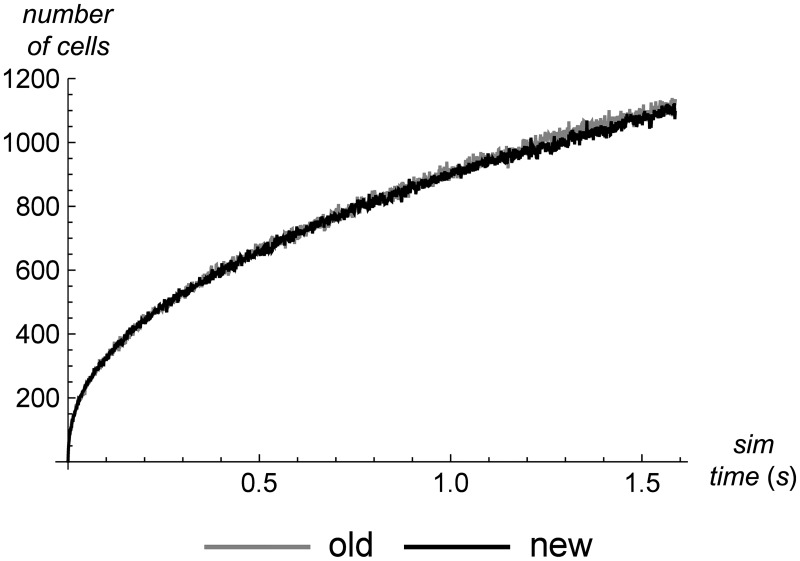
Number of cells used in old vs. new simulator. A comparison of the number of cells used in the old version of the simulator versus the new version, at the same simulation time point in the same problem (a shock tube with precision set high to create many cells). The comparison shows that the two versions use almost the name number of cells throughout the simulation, which is important to insure that performance tests are comparable.

First we check the number of cells used over the course of both simulations, to be sure our performance numbers will be directly comparable across simulator versions. Fig 9 shows that the number of cells used during both simulations follows a similar trajectory, so the results that follow should reflect the true differences in the simulation algorithm, rather than some other change that alters the amount of work the simulator performs.

**Fig 10:**
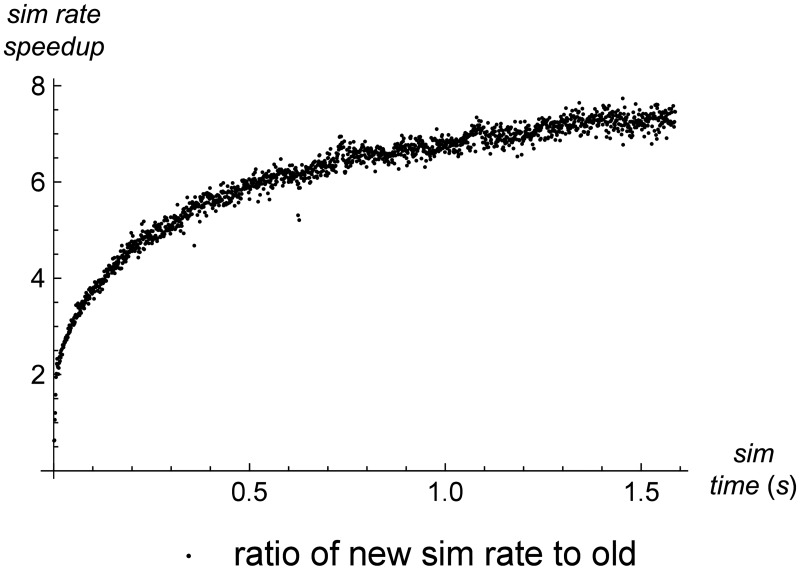
Simulation rate in old vs. new simulator. A comparison of the simulation rate achieved by the old and new versions of the simulator over the course of the test. The simulation rate is defined as simulation time (in seconds) per wall clock time (in milliseconds). We can see that once the number of cells grows sufficiently large, the new simulator achieves a rate of approximately 6x that of the old simulator.

Fig 10 shows the increase in simulation rate that the new version of the simulator achieves over the old version. We define simulation rate as the amount of simulation time we can process per unit of wall-clock time. The speedup is the ratio of the simulation rate of the new version to the simulation rate of the old version. Early in the simulation, when the number of cells is still small, both simulators are fast, and the new version shows less than a 2× speedup. But by the time the number of cells exceeds approximately 600, the new simulator is 6× as fast as the old one, and the speedup continues to increase over the course of the simulation as the number of cells increases.

**Fig 11:**
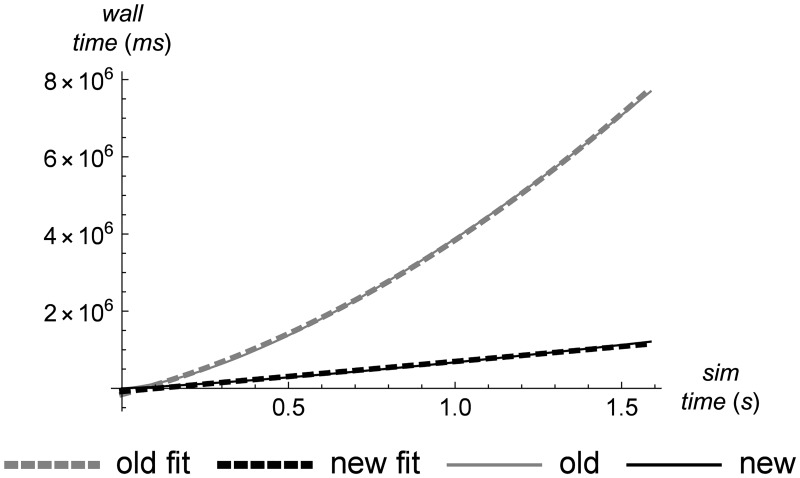
Wall-clock time as a function of simulation time. A comparison of the amount of wall-clock time required to reach a given point in simulation time. A least-squares fit of this function for the old simulator is *f*(*x*) = −155182 + 2.2971 × 10^6^ *x* + 1.70013 × 10^6^ *x*^2^, and for the new simulator is *f*(*x*) = −80049.3 + 777338*x*.

A more human-centric measure of simulation performance is how the rate of wall-clock time increase changes over the course of the simulation. Fig 11 shows that as the simulation progresses, the amount of wall-clock time required to process a unit of simulation time goes up superlinearly for the old version of the simulator, resulting in the perception that the simulation is running more and more slowly. Some slowdown is to be expected of course, since the number of cells is increasing, which requires more computational effort. But the superlinear nature of it makes this expected behavior feel worse. In contrast, this quantity goes up only linearly for the new version of the simulator.

**Fig 12:**
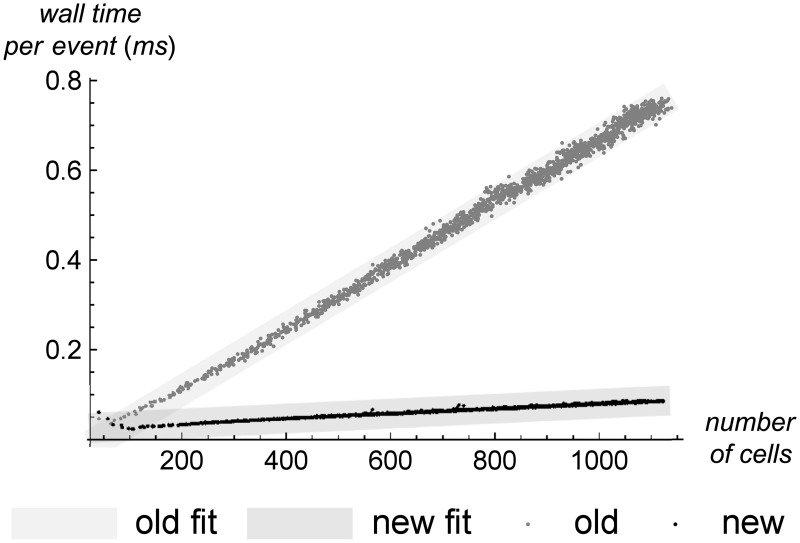
Event time vs. the number of cells. The wall-clock time required to process a simulation event, as a function of the number of cells. A least-squares fit of this function for the old simulator is *f*(*x*) = −0.0299517 + 0.000697904*x*, and for the new simulator is *f*(*x*) = 0.0246154 + 0.0000552215*x*. Both are linear, but the old simulator’s event time increases approximately 12.6× more rapidly with the number of cells.

The final performance measure we explore is how much wall-clock time is required to process each simulation event. Fig 12 plots this versus the number of cells, and we can see a clear difference between the old and new versions of the simulator. Both versions are linear in the number of cells, but the slopes differ by a factor of 12.6×, showing that the new version is considerably faster on a per-event, per-cell basis.

## Derivation of Riemann solvers

To validate our simulation results for both linear and nonlinear elasticity, we derive solvers for the Riemann problem of left and right constant states ***W***_*L*_ and ***W***_*R*_ separated by a discontinuity at *x* = 0. The primitive variables we use are either ***W*** = [*w*_1_ *w*_2_ *w*_3_]^*T*^ = [*ρ* *u* *T*]^*T*^ or [*ρ* *u* *σ*]^*T*^, where *ρ* is density in spatial coordinates, *u* is velocity, *T* is temperature, and *σ* is Cauchy stress, as discussed in the elastic equations section. We will use one set or the other of primitive variables depending on which is more mathematically convenient for a given part of the derivation, but we will always express initial conditions in terms of temperature instead of stress, because we find the temperature values more intuitive.

In this section we use the theory of quasi-linear systems of hyperbolic partial differential equations, and generally follow the notation of Toro [2] and more recently Titarev et al. [38] But this section is meant only to outline the application of this procedure to these specific elastic systems, not as a rigorous derivation of the procedure itself, so the reader is advised to consult the cited references for more detailed information.

### Introduction to the Riemann solver derivation procedure

For both the linear and nonlinear elastic systems described above, we will initially write the conservation equations in differential form

$$\begin{array}{c} U_{t}+F{\left(U\right)}_{x}=0 \end{array}$$ (14)

where the conserved variables ***U*** and the fluxes ***F***(***U***) of mass, momentum, and energy are

$$\begin{array}{c} \begin{array}{cc} U & =[\begin{array}{c} u_{1}\\ u_{2}\\ u_{3} \end{array}]=[\begin{array}{c} \rho \\ \rho u\\ \Psi_{tot}\left(\rho ,u,T\right) \end{array}]\\ F(U) & =[\begin{array}{c} f_{1}\\ f_{2}\\ f_{3} \end{array}]=[\begin{array}{c} \rho u\\ \rho u^{2}-\sigma \left(\rho ,T\right)\\ u(\Psi_{tot}\left(\rho ,u,T\right)-\sigma \left(\rho ,T\right)) \end{array}] \end{array} \end{array}$$ (15)

where definitions of Ψ_*tot*_(λ, *u*, *T*) and *σ*(λ, *T*) are given in the sections on linear and nonlinear elasticity, and we use λ = *ρ*_0_/*ρ* to transform Ψ_*tot*_(λ, *u*, *T*) to Ψ_*tot*_(*ρ*, *u*, *T*) and *σ*(λ, *T*) to *σ*(*ρ*, *T*).

To convert the system to conservative quasi-linear form

$$\begin{array}{c} U_{t}+A\left(U\right)U_{x}=0 \end{array}$$ (16)

we use the Jacobian matrix

$$\begin{array}{c} A\left(U\right)=\frac{\partial F}{\partial U}=[\begin{array}{ccc} \partial f_{1}/\partial u_{1} & \partial f_{1}/\partial u_{2} & \partial f_{1}/\partial u_{3}\\ \partial f_{2}/\partial u_{1} & \partial f_{2}/\partial u_{2} & \partial f_{2}/\partial u_{3}\\ \partial f_{3}/\partial u_{1} & \partial f_{3}/\partial u_{2} & \partial f_{3}/\partial u_{3} \end{array}] \end{array}$$ (17)

To convert the system into non-conservative quasi-linear form

$$\begin{array}{c} W_{t}+A\left(W\right)W_{x}=0 \end{array}$$ (18)

we first write down Eq (14), then evaluate the partial derivatives of the primitive variables with respect to *t* and *x* and rearrange terms to form the matrix ***A***(***W***).

Once we have ***A***(***U***) and ***A***(***W***), we can use the eigenvalues λ_*i*_ and eigenvectors ***K***_*i*_ = [*k*_*i*1_ *k*_*i*2_ *k*_*i*3_]^*T*^ of these matrices, together with the generalized Riemann invariants across rarefactions and contacts, and the Rankine-Hugoniot conditions across shocks, to derive a complete solution *f*(*x*, *t*,***W***_*L*_,***W***_*R*_) to the Riemann problem.

The solution will consist of four constant states ***W***_*L*_, W_*L_, W_*R_, ***W***_*R*_ separated by three waves which propagate at speeds given by the eigenvalues λ_1_, λ_2_, λ_3_. The left (1) and right (3) waves can be either shocks or rarefactions; the center (2) wave is always a contact. The star left state W_*L_ and star right state W_*R_ surround the contact; finding their unknown components in terms of the initial conditions ***W***_*L*_ and ***W***_*R*_ is the first part of solving a Riemann problem.

To determine if a wave *i* is a shock, a contact, or a rarefaction, we check if its characteristics are converging, parallel, or diverging, respectively, on the left (*l*) and right (*r*) sides of the wave:

$$\begin{array}{cc} \lambda_{i}\left(W_{l}\right)\geq S_{i}\geq \lambda_{i}\left(W_{r}\right) & shock \end{array}$$ (19)

$$\begin{array}{cc} \lambda_{i}\left(W_{l}\right)=S_{i}=\lambda_{i}\left(W_{r}\right) & contact \end{array}$$ (20)

$$\begin{array}{cc} \lambda_{i}\left(W_{l}\right)<\lambda_{i}\left(W_{r}\right) & rarefaction \end{array}$$ (21)

Note that contacts and degenerate shocks can both have parallel characteristics. The difference is that velocity changes across shocks, but not across contacts.

We can derive generalized Riemann invariants from the eigenvectors of the three waves:

$$\begin{array}{c} \frac{dw_{1}}{k_{i1}}=\frac{dw_{2}}{k_{i2}}=\frac{dw_{3}}{k_{i3}} \end{array}$$ (22)

These ordinary differential equations allow us to form two invariants that hold across each wave *i*. These invariants hold only for rarefactions and contacts.

A component *k*_*ij*_ of an eigenvector ***K***_*i*_ will be zero where the corresponding variable does not change across wave *i*. Note that for eigenvectors derived from non-conservative forms, this may only be true for rarefactions, depending on which set of primitive variables is chosen.

The Rankine-Hugoniot conditions express the fact that mass, momentum and energy must be conserved across shocks. They are written

$$\begin{array}{c} F\left(U_{r}\right)-F\left(U_{l}\right)=S_{i}\left(U_{r}-U_{l}\right) \end{array}$$ (23)

where *S*_*i*_ is the speed of the shock at characteristic *i*, and the fluxes and conserved variables are those on the left and right sides of the shock. In a coordinate system moving with the shock, the Rankine-Hugoniot relations reduce to ***F***(***U***_*l*_) = ***F***(***U***_*r*_), since the local shock speed is zero.

### Riemann solver for linear elasticity

Evaluating the Jacobian ***A***(***U***) = ∂***F***/∂***U*** for our linear elastic system, we obtain

$$\begin{array}{c} A\left(U\right)=[\begin{array}{ccc} 0 & 1 & 0\\ -u^{2}+\frac{E\rho_{0}}{\rho^{2}} & 2u & 0\\ -\frac{u(\rho^{2}\left(2C_{v}T+u^{2}\rho_{0}\right)+E\left(\rho^{2}-3\rho_{0}^{2}\right))}{2\rho^{2}\rho_{0}} & \frac{1}{2}\left(u^{2}+\frac{E+2C_{v}T-\frac{E\rho_{0}^{2}}{\rho^{2}}}{\rho_{0}}\right) & u \end{array}] \end{array}$$ (24)

The eigenvalues of this system are

$$\begin{array}{c} \lambda_{1}=u-a\;\lambda_{2}=u\;\lambda_{3}=u+a \end{array}$$ (25)

where the speed of sound $a=\sqrt{E\rho_{0}}/\rho$. This corresponds to a system with a shock or rarefaction traveling left at speed *u* − *a*, a contact moving with the material at speed *u*, and a shock or rarefaction traveling right at speed *u* + *a*.

In non-conservative form, the matrix is

$$\begin{array}{c} A\left(W\right)=[\begin{array}{ccc} u & \frac{E}{aa_{0}} & 0\\ \frac{a^{3}a_{0}}{E} & u & 0\\ 0 & 0 & u \end{array}] \end{array}$$ (26)

where *a*_0_ = *a*|_*ρ* = *ρ*_0__. The eigenvalues of this system are the same as those derived from the conservative formulation, which is a useful check. The eigenvectors of the non-conservative form are

$$\begin{array}{c} K_{1}=[\begin{array}{c} k_{11}\\ k_{12}\\ k_{13} \end{array}]=[\begin{array}{c} -\frac{E}{a^{2}a_{0}}\\ 1\\ 0 \end{array}]\;K_{2}=[\begin{array}{c} k_{21}\\ k_{22}\\ k_{23} \end{array}]=[\begin{array}{c} 0\\ 0\\ 1 \end{array}]\;K_{3}=[\begin{array}{c} k_{31}\\ k_{32}\\ k_{33} \end{array}]=[\begin{array}{c} \frac{E}{a^{2}a_{0}}\\ 1\\ 0 \end{array}] \end{array}$$ (27)

Looking at the zero components of the eigenvectors, we can see that the temperature is constant across the left and right waves, and the density and velocity are constant across the contact, which means the solution consists of the four states

$$\begin{array}{c} W_{L}=[\begin{array}{c} \rho_{L}\\ u_{L}\\ T_{L} \end{array}]\;W_{*L}=[\begin{array}{c} \rho_{*}\\ u_{*}\\ T_{L} \end{array}]\;W_{*R}=[\begin{array}{c} \rho_{*}\\ u_{*}\\ T_{R} \end{array}]\;W_{R}=[\begin{array}{c} \rho_{R}\\ u_{R}\\ T_{R} \end{array}] \end{array}$$ (28)

separated by the three waves traveling at speeds λ_1_, λ_2_, and λ_3_.

There are two ways of deriving expressions for ρ_*_ and u_*_. The first is to use the generalized Riemann invariants; the second is to use the Rankine-Hugoniot conditions. For this linear elastic system, both methods give the same result for ρ_*_ and u_*_, and our analysis of the characteristic speeds will show that the waves are always degenerate shocks, so we will only show the derivation of the Rankine-Hugoniot conditions here.

First, we transform the speeds *u*_*L*_, u_*_, and *u*_*R*_, to a coordinate system that moves with the shock *i* by using

$$\begin{array}{c} {\hat{u}}_{L}=u_{L}-S_{i}\;{\hat{u}}_{*}=u_{*}-S_{i}\;{\hat{u}}_{R}=u_{R}-S_{i} \end{array}$$ (29)

In a coordinate system moving with the shock, the Rankine-Hugoniot relations reduce to ***F***(***U***_*l*_) = ***F***(***U***_*r*_), since the local shock speed is zero. So for each wave, we have two coordinate transformation equations and three Rankine-Hugoniot equations, one each for mass, momentum, and energy. Eliminating the hatted velocities and shock speeds and solving for u_*_ for waves 1 and 3, we obtain two expressions for u_*_

$$\begin{array}{c} u_{*}=u_{L}+\frac{\sqrt{E\rho_{0}}\left(\rho_{L}-\rho_{*}\right)}{\rho_{L}\rho_{*}}=u_{R}-\frac{\sqrt{E\rho_{0}}\left(\rho_{R}-\rho_{*}\right)}{\rho_{R}\rho_{*}} \end{array}$$ (30)

Solving this for ρ_*_, we obtain

$$\begin{array}{c} \rho_{*}=\frac{2\sqrt{E\rho_{0}}\rho_{L}\rho_{R}}{\sqrt{E\rho_{0}}\left(\rho_{L}+\rho_{R}\right)+\rho_{L}\rho_{R}\left(-u_{L}+u_{R}\right)} \end{array}$$ (31)

Alternatively, using the same five equations per wave, but eliminating u_*_ and solving for shock speeds gives

$$\begin{array}{c} S_{1}=u_{L}-\frac{\sqrt{E\rho_{0}}}{\rho_{L}}=u_{L}-a_{L} \end{array}$$ (32)

$$\begin{array}{c} S_{3}=u_{R}+\frac{\sqrt{E\rho_{0}}}{\rho_{R}}=u_{R}+a_{R} \end{array}$$ (33)

which describes a left shock traveling at the speed of sound, and a right shock traveling at the speed of sound.

We can see that both waves are shocks because

$$\begin{array}{c} \lambda_{1}\left(W_{L}\right)=S_{1}=\lambda_{1}\left(W_{*}\right)=u_{L}-a_{L} \end{array}$$ (34)

$$\begin{array}{c} \lambda_{3}\left(W_{*}\right)=S_{3}=\lambda_{3}\left(W_{R}\right)=u_{R}+a_{R} \end{array}$$ (35)

Note that contacts can also have parallel characteristics, but we know these are shocks because velocity changes across them, since *u*_*L*_ ≠ u_*_ and u_*_ ≠ *u*_*R*_ in general. We call these shocks “degenerate” because they are discontinuous but travel only at the speed of sound, whereas shocks in more complex systems can travel faster than sound.

Now that we can calculate ρ_*_, u_*_, *S*_1_, and *S*_3_, this completes the solution of the Riemann problem for linear elasticity. Note that the temperature does not appear in any of these expressions, and since this solution procedure does not model diffusion across the contact, the left and right temperature states never mix. Sampling the solution requires simply finding the shock positions at the desired time *t*, then outputting the correct one of the four constant states ***W***_*L*_, W_*L_, W_*R_, or ***W***_*R*_ that corresponds to the desired spatial coordinate *x*.

### Riemann solver for nonlinear elasticity

Evaluating the Jacobian ***A***(***U***) = ∂***F***/∂***U*** for our nonlinear elastic system, we obtain

$$\begin{array}{c} A\left(U\right)=[\begin{array}{ccc} 0 & 1 & 0\\ \frac{1}{2}\left(-u^{2}+\frac{3E}{\rho_{0}}+\frac{3E\rho 0}{\rho^{2}}\right) & u & 1\\ \frac{u(-2C_{v}T\rho^{3}+E\left(-2\rho^{3}+3\rho^{2}\rho_{0}+2\rho_{0}^{3}\right))}{\rho^{2}\rho_{0}^{2}} & \frac{1}{2}\left(-u^{2}+\frac{4C_{v}T\rho +E\left(4\rho -3\rho_{0}-\frac{\rho_{0}^{3}}{\rho^{2}}\right)}{\rho_{0}^{2}}\right) & 2u \end{array}] \end{array}$$ (36)

The eigenvalues of this system are

$$\begin{array}{c} \lambda_{1}=u-a\;\lambda_{2}=u\;\lambda_{3}=u+a \end{array}$$ (37)

where the speed of sound $a=\sqrt{\frac{E\rho_{0}}{\rho^{2}}+\frac{2(E+C_{v}T)\rho }{\rho_{0}^{2}}}$. This corresponds to a system with a shock or rarefaction traveling left at speed *u* − *a*, a contact moving with the material at speed *u*, and a shock or rarefaction traveling right at speed *u* + *a*. Note that for nonlinear elasticity the speed of sound depends on *T*, which will complicate the solution process.

In non-conservative form, the matrix is

$$\begin{array}{c} A\left(W\right)=[\begin{array}{ccc} u & \rho & 0\\ \frac{a^{2}}{\rho } & u & \frac{C_{v}\rho }{\rho_{0}^{2}}\\ 0 & 0 & u \end{array}] \end{array}$$ (38)

The eigenvalues of this system are the same as those derived from the conservative formulation, which is a useful check. The eigenvectors are

$$\begin{array}{c} K_{1}=[\begin{array}{c} k_{11}\\ k_{12}\\ k_{13} \end{array}]=[\begin{array}{c} -\frac{\rho }{a}\\ 1\\ 0 \end{array}]\;K_{2}=[\begin{array}{c} k_{21}\\ k_{22}\\ k_{23} \end{array}]=[\begin{array}{c} -\frac{C_{v}\rho^{2}}{a^{2}\rho_{0}^{2}}\\ 0\\ 1 \end{array}]\;K_{3}=[\begin{array}{c} k_{31}\\ k_{32}\\ k_{33} \end{array}]=[\begin{array}{c} \frac{\rho }{a}\\ 1\\ 0 \end{array}] \end{array}$$ (39)

Looking at the zero components of the eigenvectors, it appears that the temperature is constant across the left and right waves, and the velocity is constant across the contact, which means that the solution would consist of the four states

$$\begin{array}{c} W_{L}=[\begin{array}{c} \rho_{L}\\ u_{L}\\ T_{L} \end{array}]\;W_{*L}=[\begin{array}{c} \rho_{*L}\\ u_{*}\\ T_{L} \end{array}]\;W_{*R}=[\begin{array}{c} \rho_{*R}\\ u_{*}\\ T_{R} \end{array}]\;W_{R}=[\begin{array}{c} \rho_{R}\\ u_{R}\\ T_{R} \end{array}] \end{array}$$ (40)

separated by the three waves λ_1_, λ_2_, and λ_3_.

However, it turns out this is only true for rarefactions. If we redo the above eigensystem with the other set of primitive variables ***W*** = [*ρ* *u* *σ*]^*T*^ by using *σ*(*ρ*, *T*) to eliminate *T* in favor of *σ*, we obtain

$$\begin{array}{c} A\left(W\right)=[\begin{array}{ccc} u & \rho & 0\\ 0 & u & \frac{-1}{\rho }\\ 0 & -a^{2}\rho & u \end{array}] \end{array}$$ (41)

which has the eigenvectors

$$\begin{array}{c} K_{1}=[\begin{array}{c} k_{11}\\ k_{12}\\ k_{13} \end{array}]=[\begin{array}{c} -\frac{1}{a^{2}}\\ \frac{1}{a\rho }\\ 1 \end{array}]\;K_{2}=[\begin{array}{c} k_{21}\\ k_{22}\\ k_{23} \end{array}]=[\begin{array}{c} 1\\ 0\\ 0 \end{array}]\;K_{3}=[\begin{array}{c} k_{31}\\ k_{32}\\ k_{33} \end{array}]=[\begin{array}{c} -\frac{1}{a^{2}}\\ -\frac{1}{a\rho }\\ 1 \end{array}] \end{array}$$ (42)

which imply a solution consisting of the four states

$$\begin{array}{c} W_{L}=[\begin{array}{c} \rho_{L}\\ u_{L}\\ \sigma_{L} \end{array}]\;W_{*L}=[\begin{array}{c} \rho_{*L}\\ u_{*}\\ \sigma_{*} \end{array}]\;W_{*R}=[\begin{array}{c} \rho_{*R}\\ u_{*}\\ \sigma_{*} \end{array}]\;W_{R}=[\begin{array}{c} \rho_{R}\\ u_{R}\\ \sigma_{R} \end{array}] \end{array}$$ (43)

This is the more general solution structure, since it allows for differing temperatures T_*L_ and T_*R_ inside shocks, and it conveniently incorporates the continuity of stress across the contact, so we will use this structure below.

In the linear elastic case, we had only two unknowns ρ_*_ and u_*_. In the nonlinear elastic case we have four: ρ_*L_, ρ_*R_, u_*_ and σ_*_. Our solution strategy will be to create two functions u_*_ = *f*_*L*_(σ_*_,***W***_*L*_) = *f*_*R*_(σ_*_,***W***_*R*_) which we will equate and solve iteratively for σ_*_. There will be one form of u_*_ = *f*(σ_*_,***W***) for rarefactions, which we will derive from the generalized Riemann invariants, and another for shocks, which we will derive from the Rankine-Hugoniot conditions.

#### Star region velocity function for rarefactions

Writing down the generalized Riemann invariants using the eigenvectors from the first non-conservative form and integrating, we have

$$\begin{array}{c} \begin{array}{ccc} & \int \frac{k_{12}}{k_{11}}\;\text{d}\rho =\int \text{d}u\; & \int k_{13}\;\text{d}u=\int k_{12}\;\text{d}T\\ & \int \frac{k_{22}}{k_{21}}\;\text{d}\rho =\int \text{d}u\; & \int k_{23}\;\text{d}u=\int k_{22}\;\text{d}T\\ & \int \frac{k_{32}}{k_{31}}\;\text{d}\rho =\int \text{d}u\; & \int k_{33}\;\text{d}u=\int k_{32}\;\text{d}T \end{array} \end{array}$$ (44)

which yield the invariants

$$\begin{array}{c} \begin{array}{ccc} & I_{11}=u+\int \frac{a}{\rho }\;\text{d}\rho \; & I_{12}=T\\ & I_{21}=u\; & I_{22}=u\\ & I_{31}=u-\int \frac{a}{\rho }\;\text{d}\rho \; & I_{32}=T \end{array} \end{array}$$ (45)

Invariants *I*_12_, *I*_21_, *I*_22_, and *I*_32_ are obvious by inspection of zeros in the eigenvectors; the generalized Riemann invariants just confirm them. Invariants *I*_11_ and *I*_31_ can be solved in terms of elliptic functions, but we leave them unevaluated for brevity. In our Riemann solver, we evaluate them by converting the integral to an initial value problem of an ordinary differential equation

$$\begin{array}{c} \frac{dy(\rho )}{d\rho }=\frac{a(\rho )}{\rho }\;y\left(1\right)=0 \end{array}$$ (46)

and integrating numerically. Applying invariants *I*_11_ and *I*_31_ across the left and right waves and solving for u_*_, we obtain

$$\begin{array}{c} u_{*L}=u_{L}+\int \frac{a(\rho_{L},T_{L})}{\rho }\;\text{d}\rho -\int \frac{a(\rho_{*L},T_{L})}{\rho }\;\text{d}\rho \end{array}$$ (47)

$$\begin{array}{c} u_{*R}=u_{R}-\int \frac{a(\rho_{R},T_{R})}{\rho }\;\text{d}\rho +\int \frac{a(\rho_{*R},T_{R})}{\rho }\;\text{d}\rho \end{array}$$ (48)

#### Star region velocity function for shocks

Applying the same velocity-transforming Rankine-Hugoniot procedure as we did for linear elasticity, we get

$$\begin{array}{c} \begin{array}{ccc} r_{1} & = & E\left(\rho_{0}^{3}-\rho_{L}^{3}\right)-\rho_{L}\left(C_{v}T_{L}\rho_{L}^{2}+\rho_{0}^{2}\sigma_{*}\right)\\ r_{2} & = & 5E\rho_{0}^{3}+E\rho_{L}^{3}+C_{v}T_{L}\rho_{L}^{3}-3\rho_{0}^{2}\rho_{L}\sigma_{*}\\ r_{3} & = & E^{2}\left(\rho_{0}^{6}+34\rho_{0}^{3}\rho_{L}^{3}+\rho_{L}^{6}\right)\\ r_{4} & = & {\left(C_{v}T_{L}\rho_{L}^{3}-3\rho_{0}^{2}\rho_{L}\sigma_{*}\right)}^{2}\\ r_{5} & = & C_{v}T_{L}\rho_{L}^{3}\left(17\rho_{0}^{3}+\rho_{L}^{3}\right)\\ r_{6} & = & 2E(r_{5}-3\rho_{0}^{2}\rho_{L}\left(\rho_{0}^{3}+\rho_{L}^{3}\right)\sigma_{*})\\ u_{*L} & = & u_{L}-\frac{1}{\sqrt{6}}\sqrt{\frac{r_{1}\left(r_{2}-\sqrt{r_{3}+r_{4}+r_{6}}\right)}{E\rho_{0}^{5}\rho_{L}^{2}}} \end{array} \end{array}$$ (49)

with a similar expression for u_*R_ which merely substitutes *R* for *L* and reverses the sign on the right-hand term.

#### Root finder for star region velocity and stress

We now have the ingredients to write an implicit equation u_*L_ = u_*R_ across the star region for the four possible solution cases: left shock/right shock, left shock/right rarefaction, left rarefaction/right shock, and left rarefaction/right rarefaction. We solve this implicit equation using a numerical root finder, whose result is a value of σ_*_ which makes u_*_ invariant across the contact. During the root finding process, we choose the shock or rarefaction u_*_ function on each side depending on whether the characteristics converge or diverge, respectively. Analysis of the shape of the u_*_ function shows that it has cusps at σ_*_ = *σ*_*L*_ and σ_*_ = *σ*_*R*_ which must not be stepped across during root finding, but the function is otherwise tractable, sloping upward for increasing σ_*_ with no local maxima or minima.

#### Finding star region densities and shock speeds

The root finder gives us values of u_*_ and σ_*_. To find ρ_*L_ and ρ_*R_ for rarefactions, we use the fact that the temperature is constant across rarefactions, as seen from the zeros of the eigenvectors. If we solve for temperature in the stress equation, and equate it across a left rarefaction, we obtain

$$\begin{array}{c} \frac{E\left(\rho_{0}^{3}-\rho_{*L}^{3}\right)-\rho_{*L}\rho_{0}^{2}\sigma_{*}}{C_{v}\rho_{*L}^{3}}=\frac{E\left(\rho_{0}^{3}-\rho_{L}^{3}\right)-\rho_{L}\rho_{0}^{2}\sigma_{L}}{C_{v}\rho_{L}^{3}} \end{array}$$ (50)

This can be solved explicitly for ρ_*L_ as a cubic in σ_*_, but this solution is numerically problematic because it involves complex-valued intermediate calculations, though the result is always real. So instead, we solve it numerically, which is simplified by the fact that *T* is a smooth function of *σ*. The solution for a right rarefaction is the same, but with *R* substituted for *L*.

For shocks, we use the Rankine-Hugoniot equations again, but this time solving for ρ_*L_ and ρ_*R_ since we have the values of u_*_ and σ_*_ available. The result is

$$\begin{array}{c} \rho_{*L}=\frac{\rho_{L}(C_{v}\rho_{L}^{3}T_{L}+E(\rho_{L}^{3}-\rho_{0}^{3})+\rho_{0}^{2}\rho_{L}\sigma_{*})}{\rho_{L}(\rho_{L}(C_{v}\rho_{L}T_{L}+\rho_{0}^{2}{\left(u_{L}-u_{*}\right)}^{2})+\rho_{0}^{2}\sigma_{*})+k(\rho_{L}^{3}-\rho_{0}^{3})} \end{array}$$ (51)

for the left side, with ρ_*R_ the same but with *R* substituted for *L*.

Once ρ_*_ is known for a shock, we can use the Rankine-Hugoniot equations one final time, this time solving for the left shock speed *S*_1_ and the right shock speed *S*_3_:

$$\begin{array}{c} S_{1}=u_{L}-\frac{\sqrt{\left(3\rho_{L}-\rho_{*L}\right)(4C_{v}\rho_{L}^{3}\rho_{*L}T_{L}+E(\rho_{0}^{3}\left(3\rho_{L}-\rho_{*L}\right)+4\rho_{L}^{3}\rho_{*L}))}}{\rho_{0}\rho_{L}\left(3\rho_{L}-\rho_{*L}\right)} \end{array}$$ (52)

$$\begin{array}{c} S_{3}=u_{R}+\frac{\sqrt{\left(3\rho_{R}-\rho_{*R}\right)(4C_{v}\rho_{R}^{3}\rho_{*R}T_{R}+E(\rho_{0}^{3}\left(3\rho_{R}-\rho_{*R}\right)+4\rho_{R}^{3}\rho_{*R}))}}{\rho_{0}\rho_{R}\left(3\rho_{R}-\rho_{*R}\right)} \end{array}$$ (53)

#### Solutions inside rarefactions

Once we have values for ρ_*L_, ρ_*R_, u_*_, and σ_*_, the last step is to derive expressions for *ρ* and *u* inside the rarefactions, since they change smoothly over some distance instead of discontinuously as in shocks. We use the fact that all characteristics start at point (*x*, *t*) = (0, 0) in the *x*-*t* plane. This means the slope of any characteristic on the left side is $\frac{x}{t}=u-a$, and on the right side is $\frac{x}{t}=u+a$. Using these equations and invariants *I*_11_ and *I*_31_ from Eq (45), we obtain for the left and right sides

$$\begin{array}{cc} & \frac{x}{t}=u_{L}-a\left(\rho ,T_{L}\right)-\int \frac{a(\rho ,T_{L})}{\rho }\;\text{d}\rho +\int \frac{a(\rho_{L},T_{L})}{\rho }\;\text{d}\rho \end{array}$$ (54)

$$\begin{array}{cc} & \frac{x}{t}=u_{R}+a\left(\rho ,T_{R}\right)+\int \frac{a(\rho ,T_{R})}{\rho }\;\text{d}\rho -\int \frac{a(\rho_{R},T_{R})}{\rho }\;\text{d}\rho \end{array}$$ (55)

We solve these using a root finder to determine *ρ* for a given point $\frac{x}{t}$. Then we substitute that *ρ* into these equations derived from the same invariants to get *u* at the same point

$$\begin{array}{cc} & u=u_{L}-\int \frac{a(\rho ,T_{L})}{\rho }\;\text{d}\rho +\int \frac{a(\rho_{L},T_{L})}{\rho }\;\text{d}\rho \end{array}$$ (56)

$$\begin{array}{cc} & u=u_{R}+\int \frac{a(\rho ,T_{R})}{\rho }\;\text{d}\rho -\int \frac{a(\rho_{R},T_{R})}{\rho }\;\text{d}\rho \end{array}$$ (57)

With this, the Riemann solver for nonlinear elasticity is complete. As in the case of linear elasticity, for any given point $\frac{x}{t}$, we first determine which characteristics it falls between, then sample the appropriate part of the solution. Note that this solver is somewhat numerically intensive, since it nests root finders and integrators inside of root finders. This is acceptable here, since our goal is merely to validate another numerical scheme with it. But this solver would likely be too inefficient to incorporate inside another scheme such as a Godunov method.

## Derivation of a Sedov-Taylor solver

A Sedov-Taylor blast wave [39] [40] [41] is a blast wave formed by placing a large amount of energy in a very small area of a stationary field whose ambient density and temperature are small by comparison. Later in the results section we will present a test problem of this type as a supplement to the other Riemann test problems.

The solution of this type of blast wave is symmetric around the blast center, and consists of two very strong diverging shocks separated by two rarefactions. We can solve for the shock positions and post-shock primitive values by using the strong-shock assumption that the speed of sound *a*_*u*_ = *a*_*L*_ = *a*_*R*_ in the unshocked material is much less than the shock speed *S*_*L*_ = *S*_*R*_. The solution for the rarefactions is more involved, and is not presented here.

We begin with the conserved variables ***U*** and the fluxes ***F***(***U***) of mass, momentum, and energy in terms of the stress *σ* instead of the temperature *T*.

$$\begin{array}{c} \begin{array}{cc} U & =[\begin{array}{c} u_{1}\\ u_{2}\\ u_{3} \end{array}]=[\begin{array}{c} \rho \\ \rho u\\ \frac{1}{2}\left(\rho u^{2}-\frac{3E\rho }{\rho_{0}}+\frac{3E\rho_{0}}{\rho }-2\sigma \right) \end{array}]\\ F(U) & =[\begin{array}{c} f_{1}\\ f_{2}\\ f_{3} \end{array}]=[\begin{array}{c} \rho u\\ \rho u^{2}-\sigma \\ \frac{1}{2}u\left(\rho u^{2}-\frac{3E\rho }{\rho_{0}}+\frac{3E\rho_{0}}{\rho }-4\sigma \right) \end{array}] \end{array} \end{array}$$ (58)

Substituting these into the Rankine-Hugoniot conditions with initial velocity *u*_*R*_ = 0, eliminating the velocity and stress variables, and taking the limit as *a*_*R*_ → 0, we obtain an expression for the right post-shock density ρ_*R_ as a function of the right initial density *ρ*_*R*_ and right shock speed *S*_*R*_:

$$\begin{array}{c} \rho_{*R}=\frac{-3E\rho_{0}\rho_{R}+3S_{R}^{2}\rho_{R}^{3}}{-3E\rho_{0}+S_{R}^{2}\rho_{R}^{2}} \end{array}$$ (59)

Similar manipulations give us the right post-shock velocity *u*_R*_

$$\begin{array}{c} u_{*R}=-\frac{2S_{R}^{3}\rho_{R}^{2}}{3E\rho_{0}-3S_{R}^{2}\rho_{R}^{2}} \end{array}$$ (60)

and right post-shock stress *σ*_R*_

$$\begin{array}{c} \sigma_{*R}=\frac{3E\rho_{0}\left(-2S_{R}^{2}\rho_{R}+\sigma_{R}\right)+S_{R}^{2}\rho_{R}^{2}\left(2S_{R}^{2}\rho_{R}+\sigma_{R}\right)}{3E\rho_{0}-3S_{R}^{2}\rho_{R}^{2}} \end{array}$$ (61)

By symmetry, the left post-shock values are the same as on the right, but with the sign of the velocity reversed.

To get the shock speed *S*_*R*_, we first find the radius of the blast wave *R*(*t*) by dimensional analysis. Our initial values are the density of the unshocked field *ρ*_*u*_ = *ρ*_*L*_ = *ρ*_*R*_ and the total energy *E*_*c*_ deposited at the blast center. The unshocked velocity *u*_*u*_ = *u*_*L*_ = *u*_*R*_ is zero, and the unshocked stress *σ*_*u*_ = *σ*_*L*_ = *σ*_*R*_ is assumed to be small compared to that of the blast. So the dimensional quantities available to us are

$$\begin{array}{ccc} \left[\rho_{u}\right] & = & \frac{M}{L}\\ \left[E_{c}\right] & = & M\frac{L^{2}}{T^{2}}\\ \left[t\right] & = & T \end{array}$$ (62)

We can then construct a quantity with the dimension of length

$$\begin{array}{c} \left[{\left(\frac{E_{c}t^{2}}{\rho_{u}}\right)}^{\frac{1}{3}}\right]=L \end{array}$$ (63)

The final expression for the radius of the blast wave as a function of time also requires a dimensionless factor *η*_*s*_ ≈ 1 to set the scale of the result

$$\begin{array}{c} R\left(t\right)=\eta_{s}{\left(\frac{E_{c}t^{2}}{\rho_{u}}\right)}^{\frac{1}{3}} \end{array}$$ (64)

And since

$$\begin{array}{c} S_{L}\left(t\right)=S_{R}\left(t\right)=\frac{dR(t)}{dt}=\frac{2R(t)}{3t} \end{array}$$ (65)

we can substitute this shock speed into equations Eqs (59), (60) and (61) to obtain the post-shock primitive values.

## Results

Here we show the results of eleven numerical test cases. The first three tests are straightforward shock tube problems, and are used to show the general nature of RRM’s solutions and to illustrate RRM’s operation using spacetime diagrams. The fourth through tenth tests are of more specialized initial conditions which were chosen because they may cause difficulties for various other types of numerical methods. The eleventh test case shows RRM’s handling of free boundary conditions.

Where available, an analytic solution from an exact Riemann solver or a Sedov-Taylor solver is shown for comparison to the simulated solution. The derivation of these solvers is presented in a separate section. After the test cases, we analyze how the simulation error decreases as we increase the computational effort by decreasing the user-specified maximum tracer particle error metric **Δ**_*max*_.

### Nonlinear elastic shock tube

**Fig 13:**
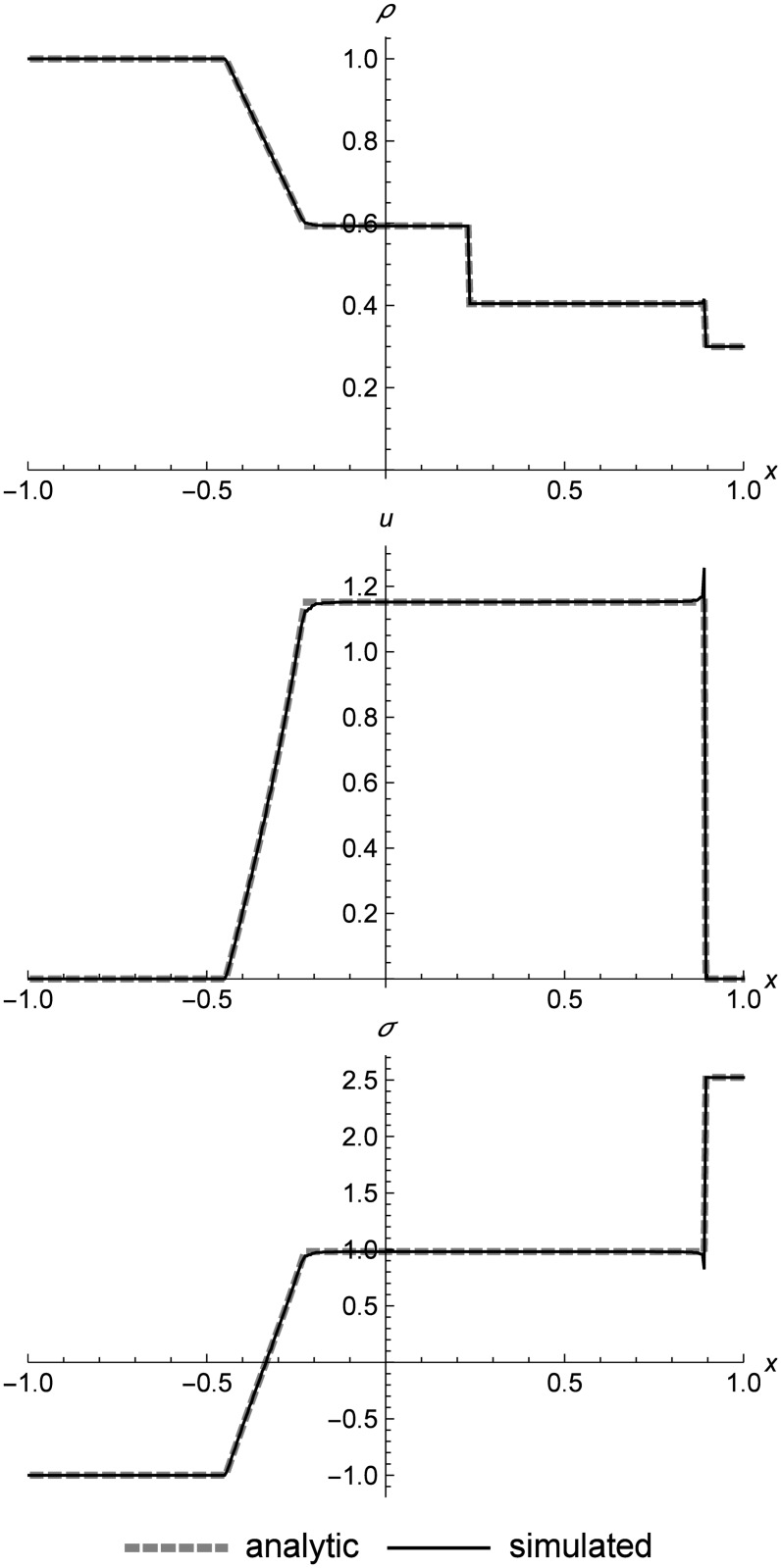
Nonlinear elastic shock tube. Two nonlinear elastic cells showing shock-tube-like evolution at *t* = 0.2. The left and right cells are designated as two different materials (with identical properties) to avoid heat diffusion across the contact, which is perfectly sharp. The velocity and stress spike at the shock is due to the fact that RRM does not incorporate a Riemann solver, so the shock speed emerges from the simulation instead of being explicitly calculated. Initial conditions are (*ρ*_*l*_, *u*_*l*_, *T*_*l*_) = (1.0, 0.0, 100.0), (*ρ*_*r*_, *u*_*r*_, *T*_*r*_) = (0.3, 0.0, 800.0), *E* = 1.0, *ρ*_0_ = 1.0, and *C*_*v*_ = 0.01. The simulation domain is *x* ∈ [−1.0, 1.0].

Fig 13 shows a shock-tube-like evolution of left rarefaction, center contact, and right shock for nonlinear elasticity. Normally the contact would be s-shaped, because RRM is not naturally adiabatic across contacts, since the replacement process mimics heat diffusion. However, in this test case we have specified in the initial conditions that the left and right cell are two different materials with identical properties, which causes flattening across contacts to produce two cells, which prevents heat diffusion and results in a sharp contact.

Note that there is a velocity and stress spike at the shock front which we do not see in the linear elastic results. This is because when we model shocks in a nonlinear elastic medium using RRM, the shock itself is a dynamic phenomenon. We do not explicitly calculate the shock speed and use it in the simulation as Godunov methods do. The tracer particles in the wavefronts spanning the shock front travel at the local speed of sound, the same as all the other tracer particles in the system. The spike is analogous to the finite thickness that real physical shocks possess, though its overshot profile differs from shocks in a real material, which are approximately sigmoidal as shown by Alsmeyer [42].

**Fig 14:**
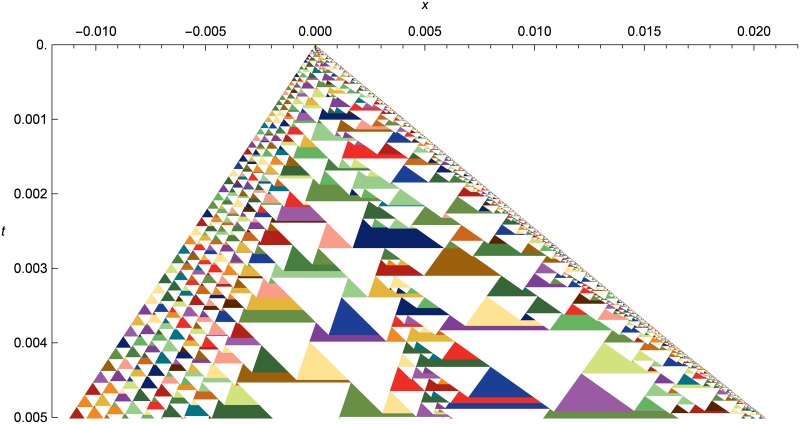
Nonlinear elastic shock tube as a spactime diagram. Shock-tube evolution from *t* = 0.0 to *t* = 0.005, with initial conditions the same as in Fig 13, but zoomed in on the center of the simulation domain. Each colored triangle is an expanding wavefront, and each white triangle is a cell. We can see the closely-spaced small triangles at left capturing the rarefaction, the line of small triangles down the middle following the contact, and the shock front using the smallest triangles at the right side.

Fig 14 shows a spacetime diagram of the shock tube problem, zoomed in to show the solution structure. Each expanding wavefront is represented by a colored triangle. When wavefronts collide and merge, the left wavefront in the merger is drawn under the right wavefront that it merges with. Any number of wavefronts may be merged together, either all at once if multiple wavefronts collide simultaneously, or successively if wavefront collisions are spread out in time.

The inverted white triangles are cells, which start at their maximum width and are progressively encompassed by the wavefronts expanding out from their left and right edges. Usually only one cell is created from a single wavefront, but along the contact, we can see single wavefronts flattening into two cells to prevent heat diffusion across the contact.

### Linear elastic double shock

**Fig 15:**
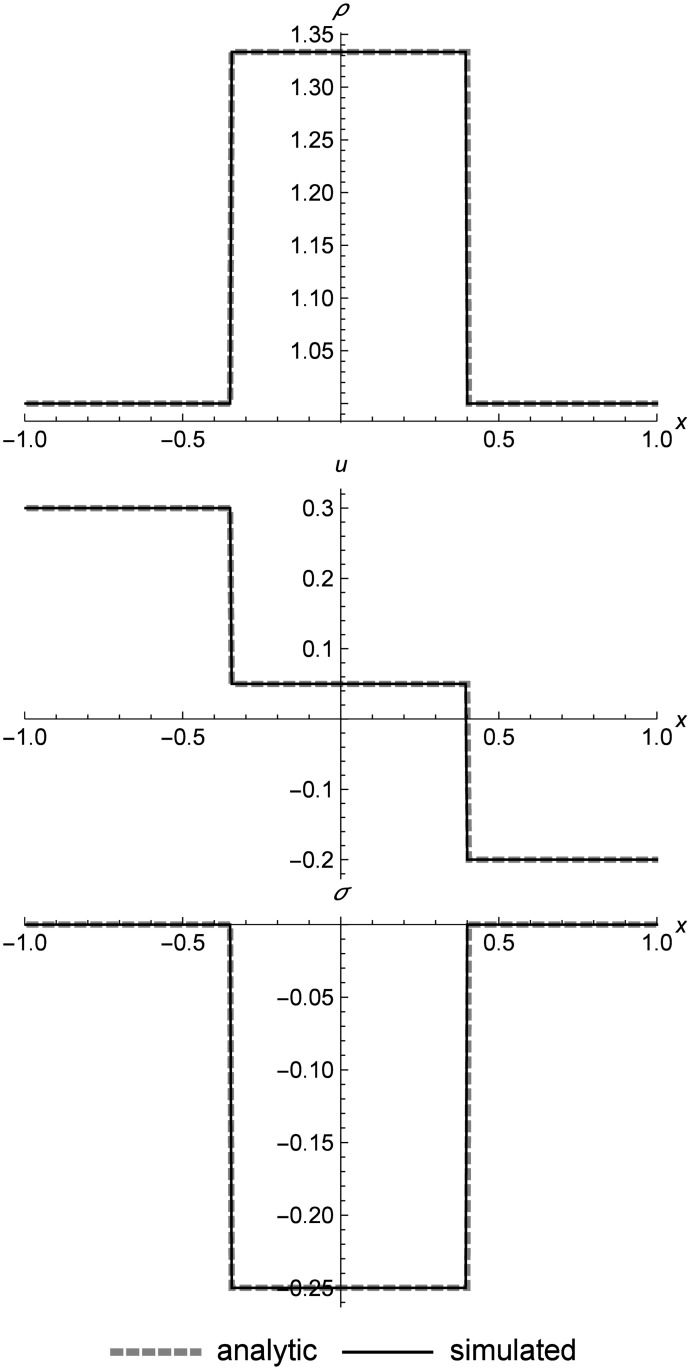
Linear elastic asymmetric double shock. Two linear elastic cells converging at different speeds to form an asymmetric double shock at *t* = 0.5. The time value is chosen to highlight (at the right shock) how the shock placement can be slightly behind the analytic value. Because the time steps in RRM only affect individual cells rather than the entire field, the shock placement will be exact only at the moments when the wavefront spanning the shock is replaced. This placement error does not accumulate over time, and can be reduced to any desired degree by reducing the user-defined error metric limit **Δ**_*max*_. Initial conditions are (*ρ*_*l*_, *u*_*l*_, *σ*_*l*_) = (1.0, 0.3, 0.0), (*ρ*_*r*_, *u*_*r*_, *σ*_*r*_) = (1.0, −0.2, 0.0), *E* = 1.0, and *ρ*_0_ = 1.0. The simulation domain is *x* ∈ [−1.0, 1.0].

Fig 15 shows an asymmetric double shock for linear elasticity. We can see that RRM keeps perfectly sharp shocks, and this is the case even for very long simulations or cases where the shocks reflect off the boundaries (which are not shown here). To prevent shocks from being erroneously merged together, we disable wavefront merging in systems like linear elasticity where we know that the solutions are always shocks, except in the case of wavefronts with very small differences between them.

One interesting peculiarity shows up on the right side of the graph, where we can see that at *t* = 0.5, the Riemann solver shows the shock slightly ahead of the simulation. This is not simply an error in shock placement. Instead, it is a reflection of the fact that RRM does not update the entire field at every time step, so the value of *t* may be slightly different for every cell. The shocks are only perfectly placed at the exact moment a replacement occurs for the wavefront spanning the shock. If desired, we can increase this replacement frequency by decreasing the user-defined error metric limit **Δ**_*max*_ of the wavefronts. Or if we wish to take a synchronized snapshot of the entire field, we could simply force all pending events to occur at the snapshot time. This placement error does not accumulate over time, so it does not affect the overall course of the simulation.

**Fig 16:**
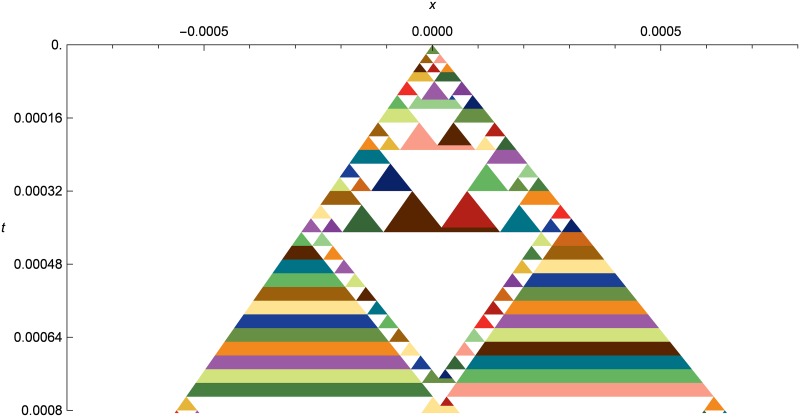
Linear elastic asymmetric double shock as a spacetime diagram. Evolution of the asymmetric double shock from *t* = 0.0 to *t* = 0.0008, with initial conditions the same as in Fig 15, but zoomed in on the center of the simulation domain. The rainbow triangles at the bottom left and right illustrate an optimization called “wavefront spanning”, where instead of creating a new cell adjacent to an older one with the same primitive values, the wavefront crossing the old cell is simply expanded to cover the new cell. This avoids creating many identical-valued cells in flat areas of shock-only systems like linear elasticity.

**Fig 17:**
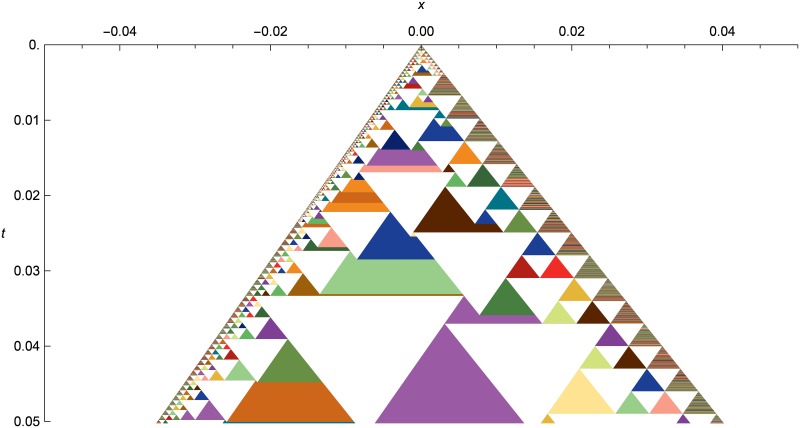
Linear elastic asymmetric double shock as a spacetime diagram (increased time span). Evolution of the asymmetric double shock from *t* = 0.0 to *t* = 0.05, with initial conditions the same as in Fig 15, but zoomed in on the center of the simulation domain. Note how the cells in the center of the flat area between the two shocks get larger and larger, whereas the cells at the shock fronts stay small to capture the time evolution at the specified resolution.

Figs 16 and 17 show spacetime diagrams of the same initial conditions, zoomed in to show the solution structure at two different levels. Note how the pattern of triangles is asymmetric, reflecting the asymmetric nature of the initial conditions. Note also the horizontally-striped “rainbow triangles” on the left and right. These illustrate an optimization called “wavefront spanning”, where we avoid creating adjacent cells with the same primitive values by expanding existing wavefronts to cover them. The expansion process creates a new wavefront and merges the old one onto it, which creates a new stripe of color since wavefronts are colored by modulo indexing into a color table using the wavefront serial number. However, a single rainbow triangle may be considered to be one expanding wavefront, albeit one where the left and right tracer particles are changed over time.

### Nonlinear elastic double rarefaction

**Fig 18:**
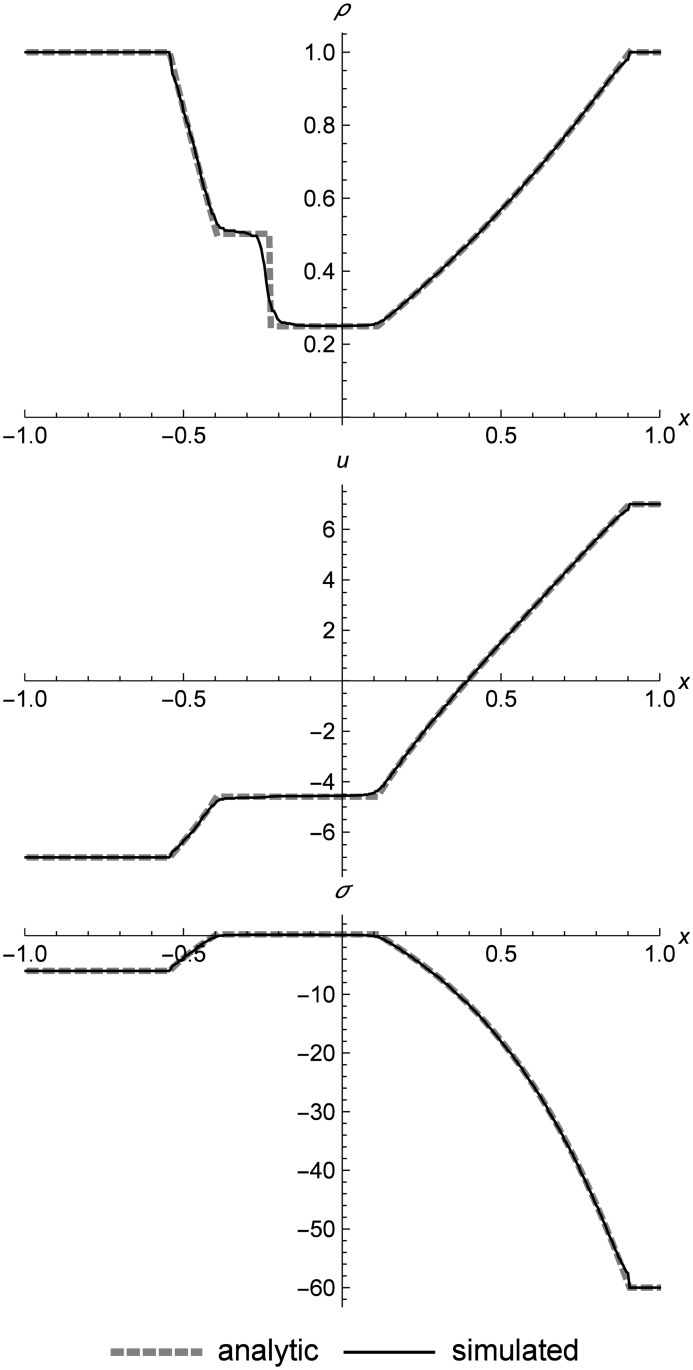
Nonlinear elastic double rarefaction. Two nonlinear elastic cells diverging to show an asymmetric double rarefaction at *t* = 0.05. This figure illustrates the s-shaped contact that is typical of RRM when we use only one material type across the entire field, which allows heat diffusion across the contact. Initial conditions are (*ρ*_*l*_, *u*_*l*_, *T*_*l*_) = (1.0, 7.0, 20.0), (*ρ*_*r*_, *u*_*r*_, *T*_*r*_) = (1.0, −7.0, 200.0), *E* = 1.0, *ρ*_0_ = 1.0, and *C*_*v*_ = 0.3. The simulation domain is *x* ∈ [−1.0, 1.0].

Fig 18 shows a left rarefaction, center contact, and right rarefaction. Here we see the s-shaped contact that is typical of RRM, since we have used only one material in the initial conditions in order to demonstrate the behavior. The complex curves of the rarefactions match the Riemann solver data closely. These initial conditions include a larger-than-normal value of *C*_*v*_ to make the test harder by increasing the step size across the contact.

**Fig 19:**
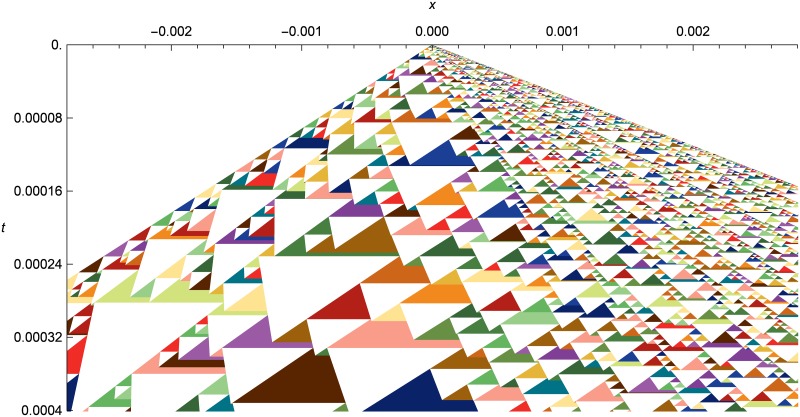
Nonlinear elastic double rarefaction as a spacetime diagram. Evolution of the asymmetric double rarefaction from *t* = 0.0 to *t* = 0.0004, with initial conditions the same as in Fig 18, but zoomed in on the center of the simulation domain. Note the adaptive distribution of cell sizes in both time and space across the diagram. Note also that the tops of the white triangles (the cells) are all horizontal, since both ends of a cell are created at the same time, but some of them appear slightly tilted due to an optical illusion.

Fig 19 shows a spacetime diagram of the same initial conditions, zoomed in to show the solution structure. Note how the cell sizes vary smoothly across both space and time in accord with the local demands of the simulation, without any sharp demarcations for submeshes or other stepwise adaptive techniques.

### Stationary contact

**Fig 20:**
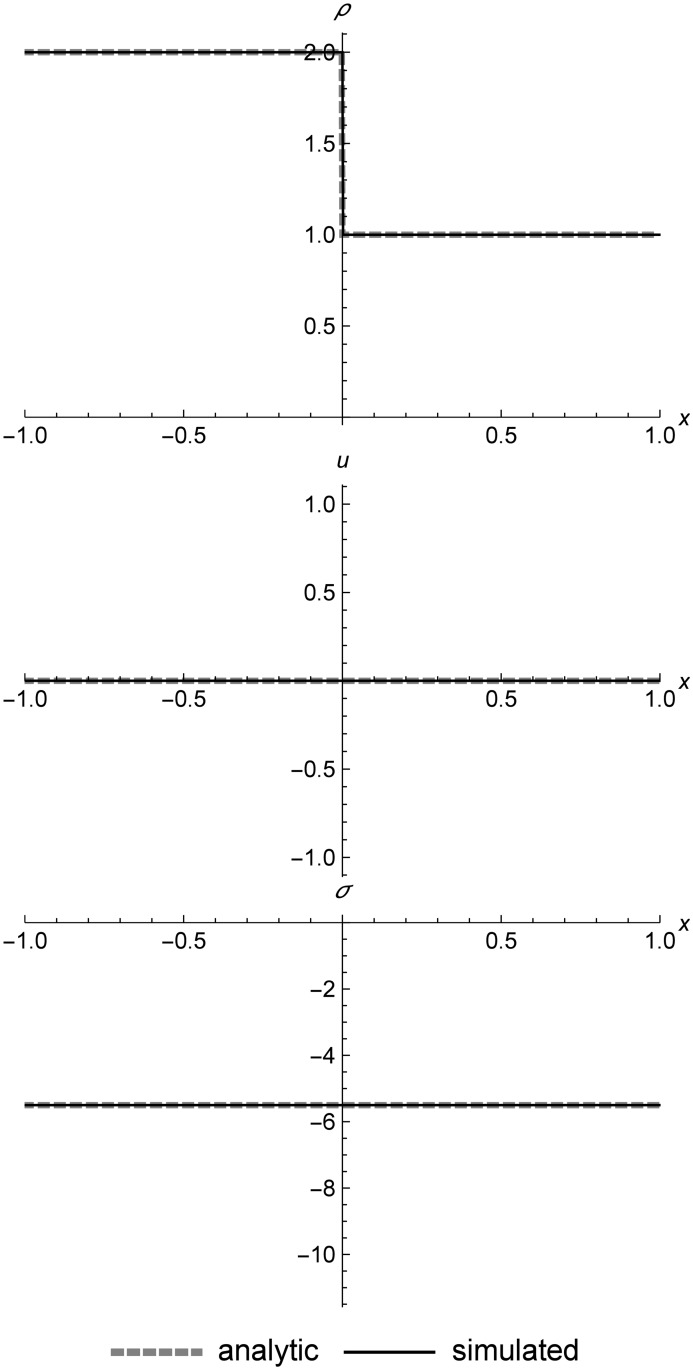
Stationary contact. A stationary contact at time *t* = 100.0. After approximately 14,000 simulation events, the contact is still perfectly sharp, since we used different material types for the left and right initial states, which prevents replacement across a contact from mixing states with different temperatures. During simulation, the domain is composed of anywhere from 2 to 12 cells. Initial conditions are (*ρ*_*l*_, *u*_*l*_, *T*_*l*_) = (2.0, 0.0, 50.0), (*ρ*_*r*_, *u*_*r*_, *T*_*r*_) = (1.0, 0.0, 550.0), *E* = 1.0, *ρ*_0_ = 1.0, and *C*_*v*_ = 0.01. The simulation domain is *x* ∈ [−1.0, 1.0].

Fig 20 shows a stationary contact in a nonlinear elastic material. These contacts are formed by finding two (*ρ*, *T*) pairs that result in identical stresses, and using those for the initial conditions. In Eulerian methods, contacts typically broaden over time due to numerical diffusion. In some Lagrangian methods such as smoothed particle hydrodynamics, contacts can change shape due to a numerical surface tension effect, depending on how those methods are implemented [43] [25] [44]. Contacts in RRM maintain their sharpness indefinitely, as long as we make the simulation adiabatic. Moving-grid methods can also maintain sharp contacts, at the cost of occasional remeshing operations when the grid becomes too deformed. RRM has the same property, but with continuous rather than occasional remeshing.

### Supersonic parallel contacts

**Fig 21:**
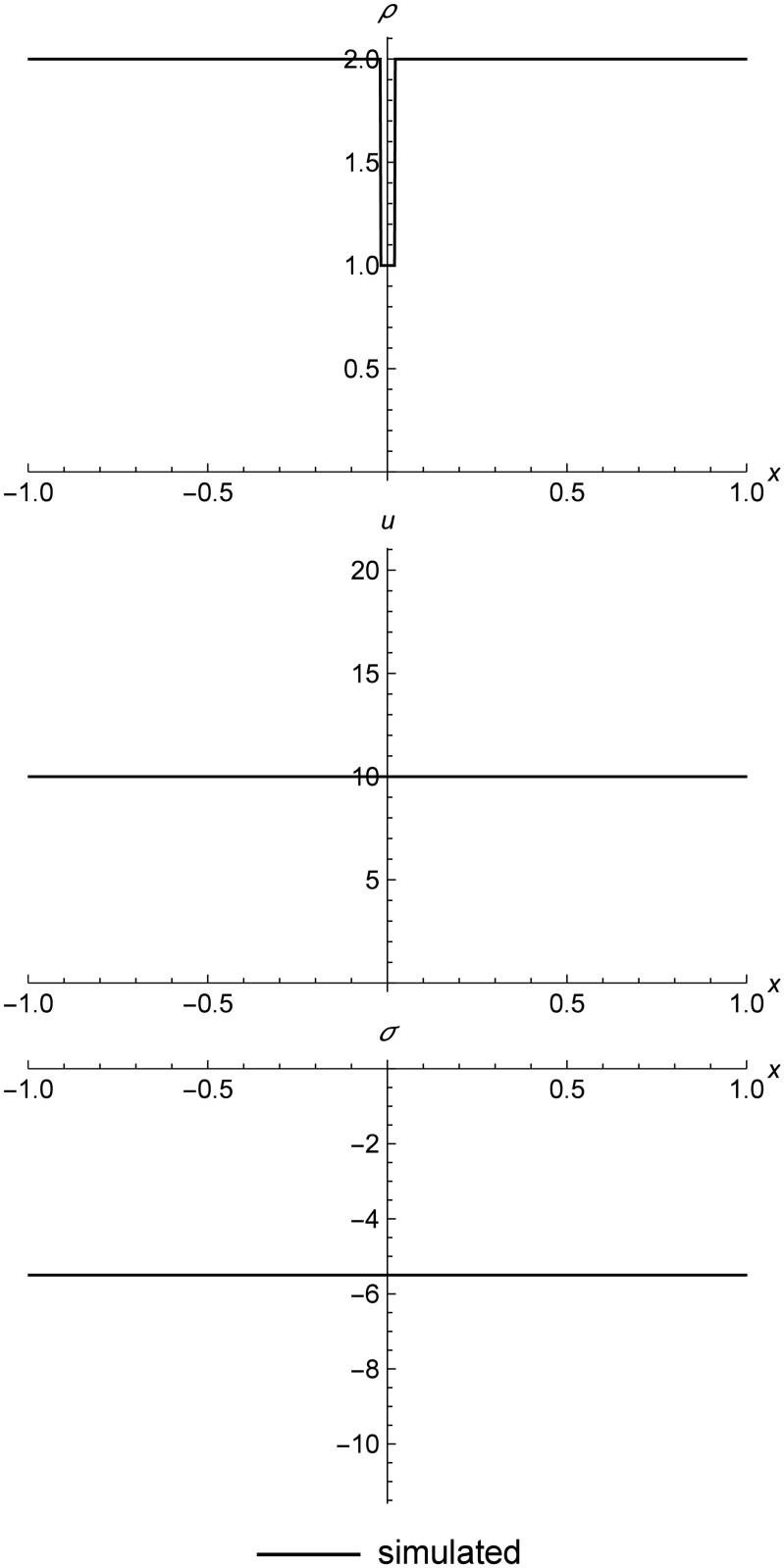
Supersonic parallel contacts. A pair of parallel contacts, moving at supersonic speed, after having traveled around the periodic simulation domain 10 times. The contacts are still sharp and separate, despite traveling at high speed and close distance from *t* = 0.0 to *t* = 200.0. During simulation, the domain is composed of anywhere from 3 to 12 cells. The speed of sound in the material ranges from *a*(*ρ*, *T*) = 2.5 to *a*(*ρ*, *T*) = 3.74. Initial conditions are (*ρ*_*h*_, *u*_*h*_, *T*_*h*_) = (2.0, 10.0, 50.0) in the high-density region (98% of the domain width), and (*ρ*_*l*_, *u*_*l*_, *T*_*l*_) = (1.0, 10.0, 550.0) in the low-density region (2% of the domain width), with *E* = 1.0, *ρ*_0_ = 1.0, and *C*_*v*_ = 0.01. The simulation domain is *x* ∈ [−1.0, 1.0] with periodic boundary conditions.

Fig 21 shows a pair of parallel contacts moving to the right at supersonic speed in a nonlinear elastic material, with Mach number in the range *M* = 2.67–4.0. Eulerian methods would typically broaden and eventually merge these contacts over time, and would require a small time step due to the supersonic velocity. RRM can advect sharp flow features at high speeds with large time steps, since the features do not have to be transferred through stationary cells of the simulation domain.

### Noh implosion

**Fig 22:**
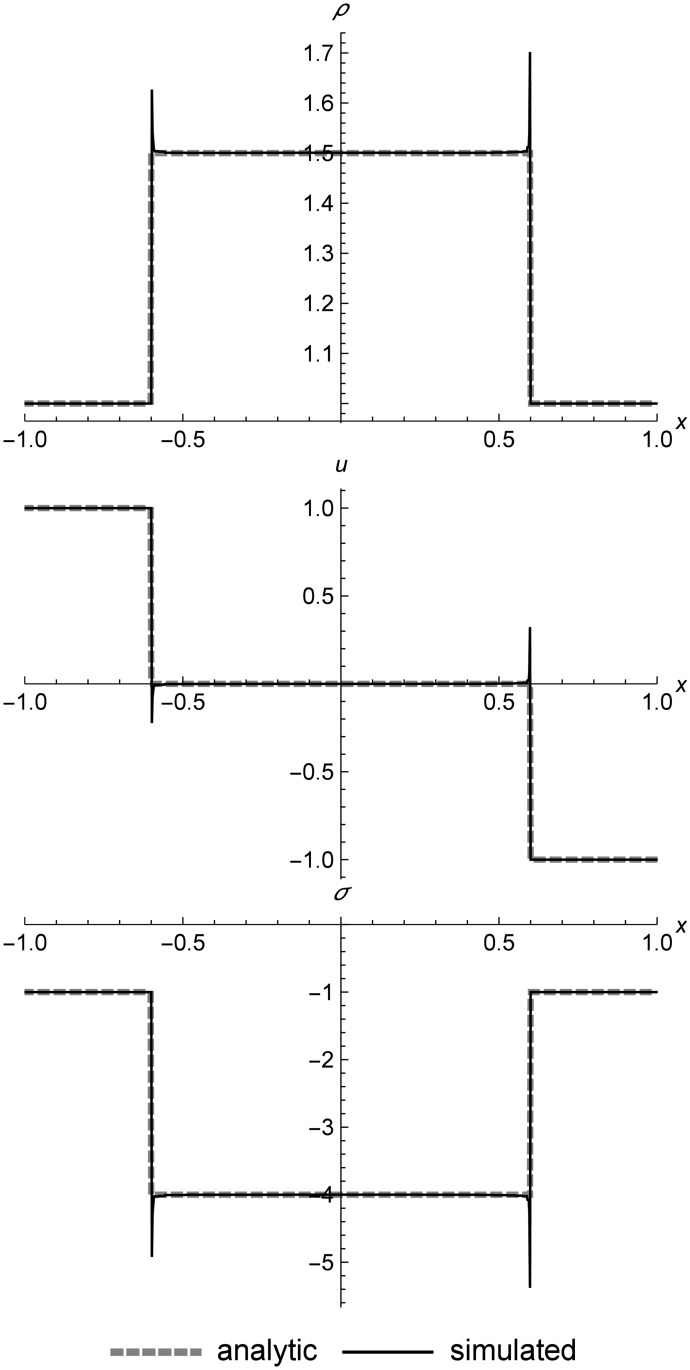
Noh implosion. A Noh implosion or impact test. Note that the solution is flat across the trivial contact in the center, which shows that RRM does not exhibit the wall-heating phenomenon that can be seen in some other Lagrangian methods. Shocks show the impulse-like overshoot typical of RRM. Initial conditions are (*ρ*_*l*_, *u*_*l*_, *T*_*l*_) = (1.0, 1.0, 100.0), (*ρ*_*r*_, *u*_*r*_, *T*_*r*_) = (1.0, −1.0, 100.0), *E* = 1.0, *ρ*_0_ = 1.0, and *C*_*v*_ = 0.01. The simulation domain is *x* ∈ [−1.0, 1.0].

Fig 22 shows a Noh implosion [45] or impact test in a nonlinear elastic material. This is a test of shock placement, symmetry, and the treatment of the trivial contact at *x* = 0.0. Even some recent and sophisticated Lagrangian techniques such as those described by Hopkins [25] and Frontiere et al. [44] can show a phenomenon known as wall-heating at the trivial contact, which causes spurious dips or peaks in the primitive variables. RRM shows accurate shock placement and no evidence of wall-heating, though it also displays a typical impulse-like overshoot at the shocks where the primitive variables transition from their pre- to post-shock values.

### Strong separation

**Fig 23:**
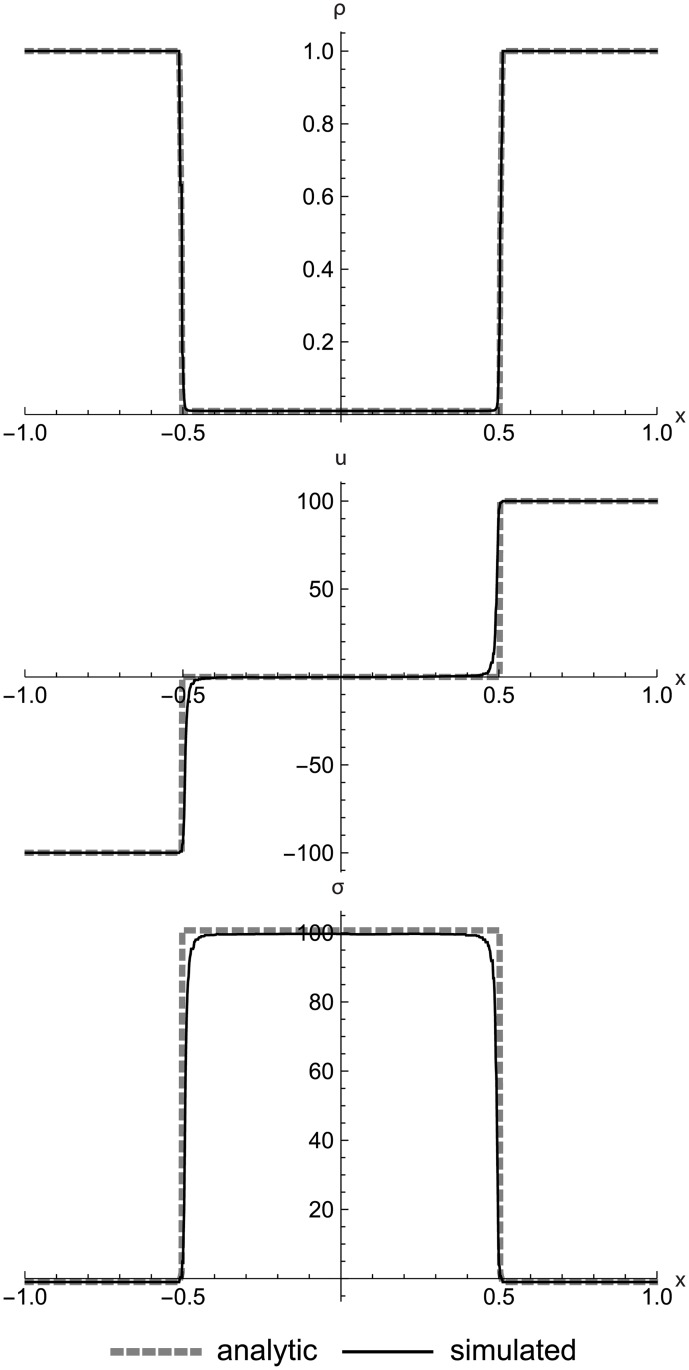
Strong separation. A strong separation test. The intent of this test is to show numerical robustness of both RRM and the nonlinear elastic Riemann solver at high stress and supersonic speed. The simulated solution is accurate except for some rounding of the inside corners. The density is pulled down to approximately 1% of its initial value at the center, but cannot be pulled to zero regardless of the separation velocity, because of the nonlinearity of the elastic material. The Mach number of the separation is *M* = 44.7. Initial conditions are (*ρ*_*l*_, *u*_*l*_, *T*_*l*_) = (1.0, −100.0, 100.0), (*ρ*_*r*_, *u*_*r*_, *T*_*r*_) = (1.0, 100.0, 100.0), *E* = 1.0, *ρ*_0_ = 1.0, and *C*_*v*_ = 0.01. The simulation domain is *x* ∈ [−1.0, 1.0].

Fig 23 shows a strong separation test, similar to the one demonstrated for a different type of nonlinear elasticity by Titarev et al. [38], but at higher separation velocity. RRM shows a flat solution across the trivial contact at *x* = 0.0, where some other methods may show dips in the primitive values at this location [38]. This test also stresses our nonlinear elastic Riemann solver, which required a more accurate numerical integrator for Eq 46 to handle these initial conditions. These initial conditions are likely beyond the range of physical validity of our constitutive equations, so we use them here merely to demonstrate numerical robustness.

### Sonic point in rarefaction

**Fig 24:**
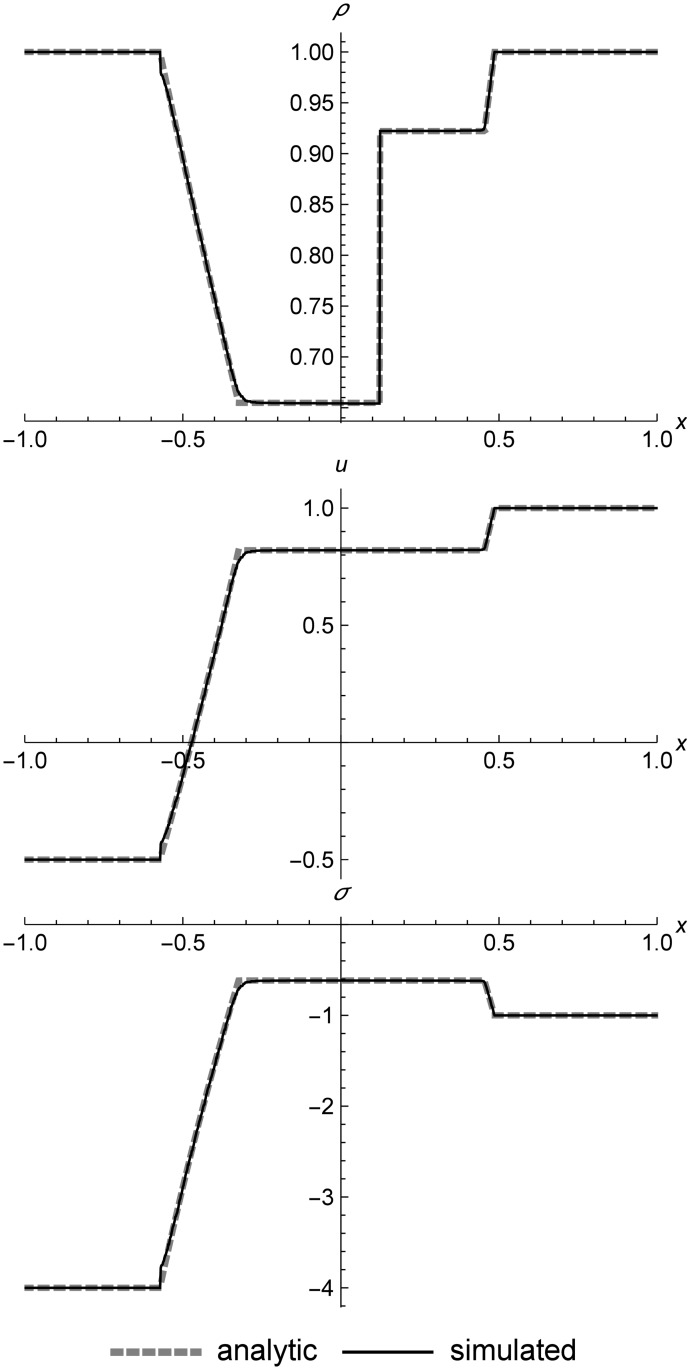
Expansive sonic point in left rarefaction. This is a test of accuracy around expansive sonic points. At *t* = 0.15, the sonic point is at *x* ≈ −0.47, inside the left rarefaction fan. We can see that there are no spurious jumps in *ρ*, *u*, or *σ* at this location. Initial conditions are (*ρ*_*l*_, *u*_*l*_, *T*_*l*_) = (1.0, −0.5, 400.0), (*ρ*_*r*_, *u*_*r*_, *T*_*r*_) = (1.0, 1.0, 100.0), *E* = 1.0, *ρ*_0_ = 1.0, and *C*_*v*_ = 0.01. The simulation domain is *x* ∈ [−1.0, 1.0].

Fig 24 shows a nonlinear elastic separation test which has been modified to have an expansive sonic point in the left rarefaction. Expansive sonic points can cause spurious rarefaction shocks in numerical methods for fluid dynamics [46], and the same can be true for nonlinear elasticity [38]. In this test case, we see that at the location of the sonic point at *x* ≈ −0.47, RRM does not produce any spurious jumps in the primitive values.

### Woodward-Colella-like blast wave

**Fig 25:**
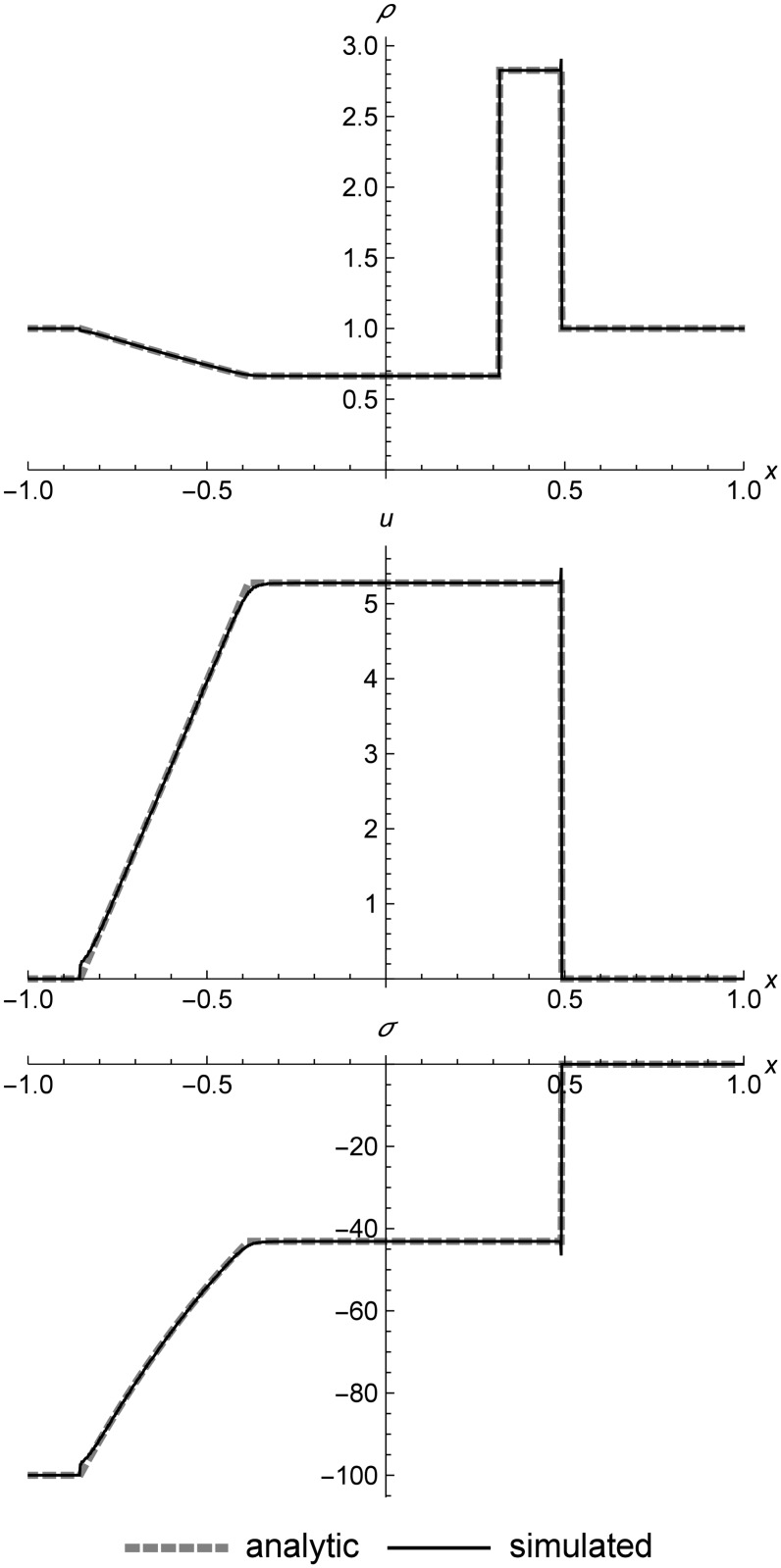
Woodward-Colella-like blast wave. A blast wave test similar to the left side of the Woodward-Colella test. This test demonstrates the ability of RRM to handle parallel contact-shock pairs moving in close proximity. Initial conditions are (*ρ*_*l*_, *u*_*l*_, *T*_*l*_) = (1.0, 0.0, 10000.0), (*ρ*_*r*_, *u*_*r*_, *T*_*r*_) = (1.0, 0.0, 1.0), *E* = 1.0, *ρ*_0_ = 1.0, and *C*_*v*_ = 0.01. The simulation domain is *x* ∈ [−1.0, 1.0].

Fig 25 shows a blast wave in a nonlinear elastic material with a temperature ratio of 10,000:1 from left to right, which is similar to the pressure ratio across the left side of the Woodward-Colella blast wave test [47]. The solution is a tall and narrow right shock, the trailing side of which is a contact moving at the same speed. This tests a numerical method’s ability to resolve parallel discontinuities of differing types, as well as general numerical robustness. RRM does well on this test, with the exception of a small overshoot at the shock front.

### Sedov-Taylor-like blast wave

**Fig 26:**
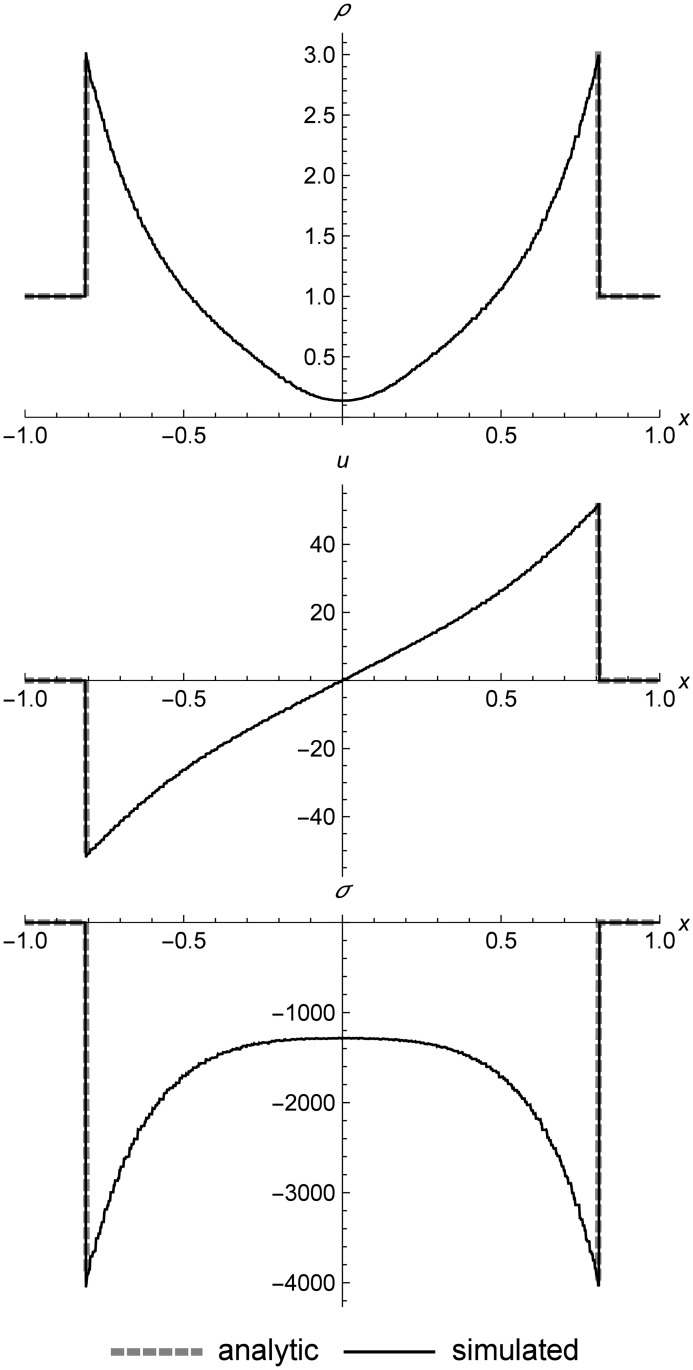
Sedov-Taylor-like blast wave. A blast wave at time *t* = 7.0 × 10^−3^ after a large amount of energy *E*_*c*_ was released at *x* = 0.0. The analytic solution plotted here shows the shock placement and the post-shock values of the primitive variables, which are in good agreement with the simulation. An analytic solution is not currently available for the interior of this problem, but the qualitative features of the interior of the simulated solution are in good agreement with the similar blast wave problem for the Euler equations. Initial conditions are (*ρ*_*c*_, *u*_*c*_, *T*_*c*_) = (1.0, 0.0, 1.0 × 10^8^) in the center region (0.2% of the domain width), and (*ρ*_*u*_, *u*_*u*_, *T*_*u*_) = (1.0, 0.0, 10.0) in the unshocked material elsewhere (99.8% of the domain width), with *E* = 1.0, *ρ*_0_ = 1.0, and *C*_*v*_ = 0.01. The simulation domain is *x* ∈ [−1.0, 1.0].

Fig 26 shows a blast wave similar to a Sedov-Taylor blast wave [39] [40] [41], but in a nonlinear elastic material instead of a gas. A large amount of energy *E*_*c*_ is placed in a narrow center region of an otherwise stationary and uniform material. These initial conditions evolve into two diverging stong shocks, separated by two rarefactions. This is a test of numerical robustness, because a numerical method must be able to convert a very concentrated energy source into a symmetrically expanding blast wave.

Comparison of the shock placement and post-shock values to an analytic solution shows good agreement. An analytic solution is not currently available for the interior of this problem, but the qualitative features of the solution are similar to those of the analytic solution for the Euler equations [44], namely the convex-upward density curve, approximately linear diverging velocity, and stress which is flat in the center. Unlike for the Euler equations, these nonlinear elastic equations do not pull the density and stress very low in the center, due to the quadratic term in the stress equation Eq (7).

### Nonlinear elastic double shock with free boundaries

**Fig 27:**
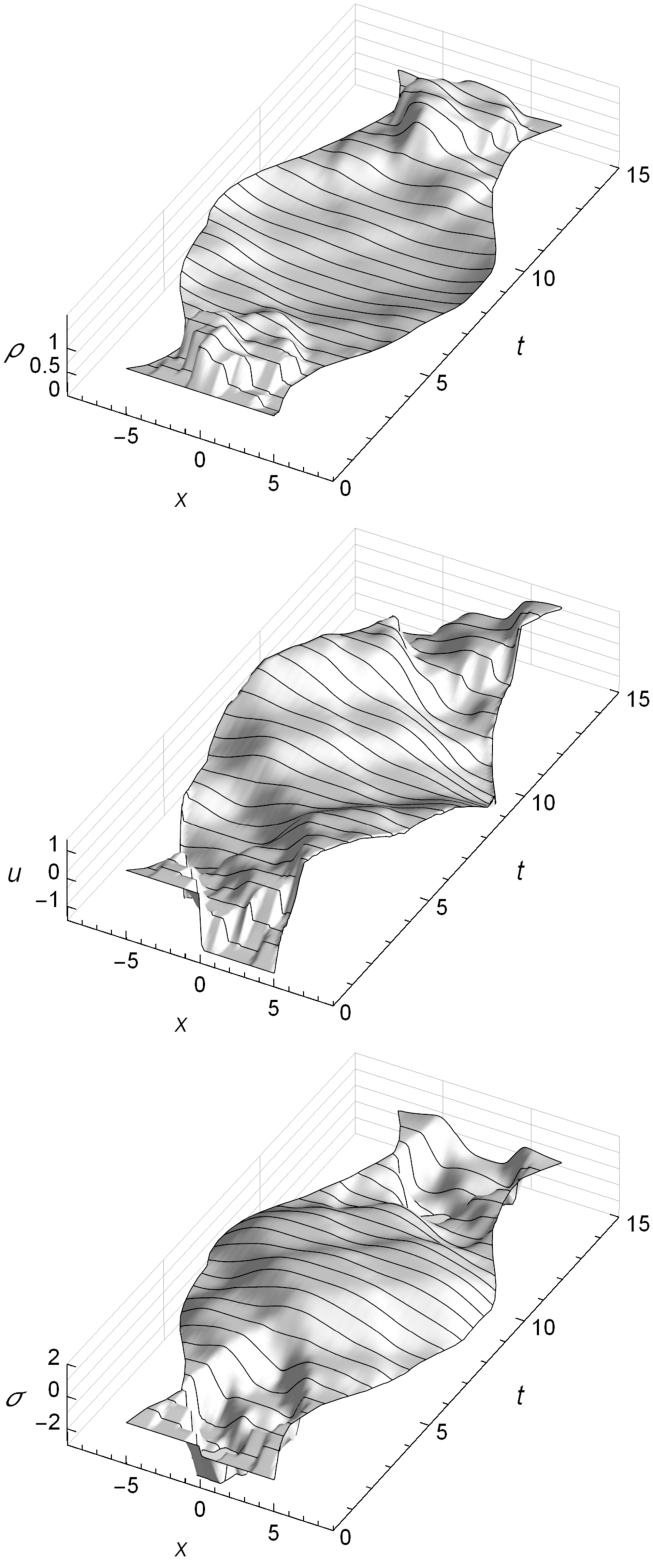
Double shock in a nonlinear elastic rod. A nonlinear elastic rod, initially made up of two cells which are converging to create an asymmetric double shock, from *t* = 0.0 to *t* = 15.0. The rod converges, rebounds out to its maximum stretched length, and converges again, showing how RRM handles free boundary conditions. The rod’s left and right edges are the left and right edges of each surface in the figure. Initial conditions are (*ρ*_*l*_, *u*_*l*_, *T*_*l*_) = (1.0, 1.0, 40.0), (*ρ*_*r*_, *u*_*r*_, *T*_*r*_) = (1.0, −1.0, 100.0), *E* = 1.0, *ρ*_0_ = 1.0, and *C*_*v*_ = 0.01. The simulation domain is *x* ∈ [−10.0, 10.0], but no work is performed in areas where there are no cells.

Fig 27 shows an asymmetric double shock in a nonlinear elastic rod with two free boundaries (the two ends of the rod). The rod initially consists of two converging cells. We can see that the length of the rod decreases initially as the two shocks propagate outwards from the center, then the rod rebounds out to its maximum stretched length at approximately *t* = 8.0, then it contracts and starts the cycle over at approximately *t* = 13.0. As the rod oscillates in and out, the shocks gradually lose their sharpness as they cross each other and reflect off the ends of the rod. Eventually the primitive values form smooth curves across the length of the rod as it continues to oscillate indefinitely.

The usual free boundary condition of an elastic substance is that *σ* = 0 at the boundaries. RRM does not need to enforce this condition explicitly. Instead, it happens naturally because at the ends of the rod, the stress momentum and stress energy are not balanced out by those of an adjacent cell, so any stress at the ends is quickly converted to kinetic energy.

### Error analysis

**Fig 28:**
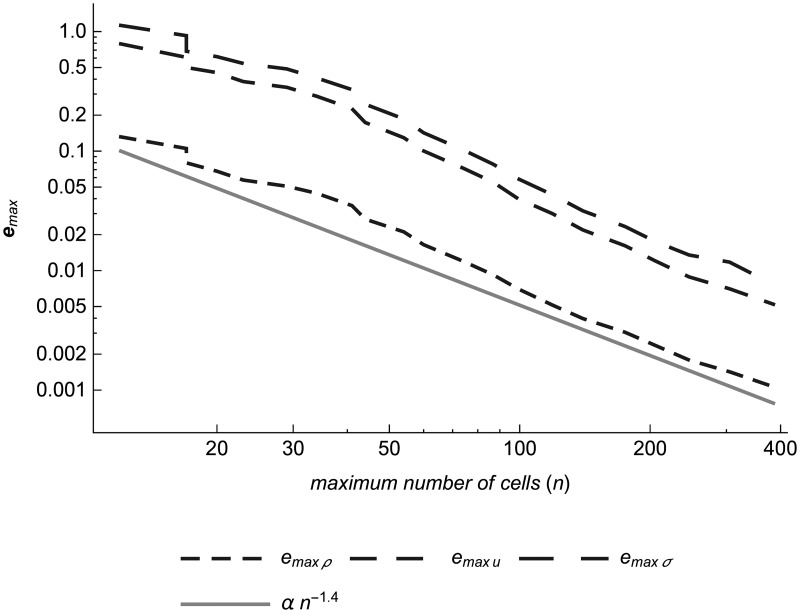
Error versus computational effort, sharp contact. The three components *e*_*maxρ*_, *e*_*maxu*_, and *e*_*maxσ*_ of the maximum integral error ***e***_*max*_ versus the maximum number of cells, for the nonlinear elastic shock tube test case from *t* = 0.0 to *t* = 0.4, with a sharp contact provided by using two material types. The error in all three primitive values is approximately proportional to *n*^−1.4^ where *n* is the maximum number of cells used in the simulation run. The value *α* is an arbitrary constant chosen to put the proportion line below the others for ease of comparison. Not shown on this graph, all three components of **Δ**_*max*_, corresponding to the user-specified error metric limit for *ρ*, *u*, and *σ*, are decreased from 10^−1^ to 10^−5^ from left to right.

If we define the point error between a simulation and a Riemann solver as ***e***(*x*, *t*) = ***W***_*RRM*_(*x*, *t*) − ***W***_*Riemann*_(*x*, *t*), then we can define a useful measure of the error of an entire simulation run as

$$\begin{array}{c} e_{max}=\max_{t}\left(\int \left|e\left(x,t\right)\right|\;\text{d}x\right) \end{array}$$ (66)

where ***e***_*max*_ is the maximum value that the space integral of the absolute value of the error takes for any time *t* during the simulation run. If we plot this quantity versus the maximum number of cells *n* used at any time during the simulation run, this will show us how the error decreases as the user decreases the tracer particle error metric limit **Δ**_*max*_, and will give us a measure of the computational requirements of the simulation. The result, shown in Fig 28, is that the error is approximately proportional to *n*^−1.4^. And since the computational effort of RRM scales as O(N), where *N* is the number of cells, the error in the simulation decreases slightly faster than linearly as we increase the computational effort.

Since RRM uses constant-valued cells, we might expect the error to decrease only linearly as we add cells. However, since the cells are not evenly spaced, but instead are concentrated in areas of higher primitive variable slope, we obtain this slightly superlinear behavior. We have not yet performed an analysis of how alternative error metrics to Eq (11) might affect this behavior.

**Fig 29:**
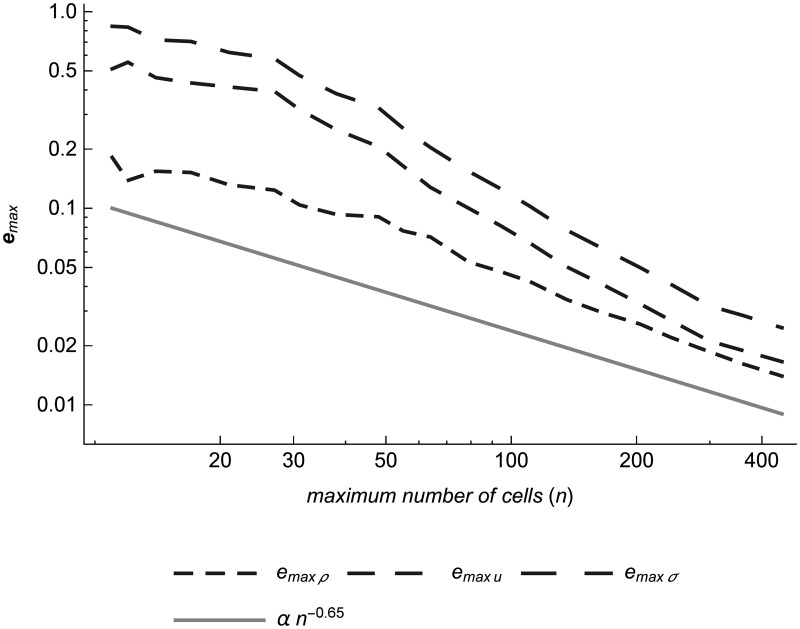
Error versus computational effort, diffusive contact. The three components *e*_*maxρ*_, *e*_*maxu*_, and *e*_*maxσ*_ of the maximum integral error ***e***_*max*_ versus the maximum number of cells, for the nonlinear elastic shock tube test case from *t* = 0.0 to *t* = 0.4, with a diffusive contact. The error in all three primitive values is approximately proportional to *n*^−0.65^ where *n* is the maximum number of cells used in the simulation run. This is significantly sublinear because without sharp contacts turned on, RRM is unable to reduce the difference between its solution and that of the Riemann solver around the contact. Note that the density component of the error *e*_*maxρ*_ is the worst of the three, since the density is what changes across a contact. The value *α* is an arbitrary constant chosen to put the proportion line below the others for ease of comparison. Not shown on this graph, all three components of **Δ**_*max*_, corresponding to the user-specified error metric limit for *ρ*, *u*, and *σ*, are decreased from 10^−1^ to 10^−5^ from left to right.

To see what RRM’s error behavior looked like before its improvements for this paper, we redo the error analysis on the same test case, but with a diffusive (non-adiabatic) contact instead of a sharp contact. This gives the results shown in Fig 29. Note that ***e***_*max*_, shown by the three dashed lines, shows approximately 10x less reduction at 400 cells than it did in the sharp contact case. In particular, the density component of the error *e*_*maxρ*_ is much worse than before. This is because in RRM, diffusive contacts converge to a sigmoidal density curve, not a step, so RRM is unable to reduce the difference between its solution and that of the Riemann solver. And since all the error reduction must come from the non-contact parts of the domain, the system must work much harder, which is reflected in a sublinear behavior where the error is approximately proportional to *n*^−0.65^.

## Summary and conclusions

We have shown that RRM performs well on problems in linear and nonlinear elasticity, giving results whose shocks and rarefactions match the Riemann and Sedov-Taylor solvers, with a well-behaved error that decreases somewhat superlinearly with increased computational effort. We have also shown how to motivate and construct a simple constitutive equation for nonlinear elasticity, and have demonstrated in the derivation section how to create Riemann solvers for both types of elastic system.

The derivative-free, solver-free nature of RRM was chosen for robustness when simulating mathematically inconvenient systems. Now that RRM has been verified to work correctly for the Euler equations, linear elasticity, and nonlinear elasticity, future work on RRM will concentrate on systems for which no analytic solution is known.

In addition to application to new systems, future work is also possible in extending the generality and speed of RRM within existing systems. This work is described below.

### Extension to 2D and 3D

The current implementation of RRM models cells as finite elements. As simulation progresses, these elements are chopped up and replaced with new cells. This approach could be directly extended to higher dimensions, but it would likely prove cumbersome to implement, since it would require cells to increase in geometric complexity as they are chopped by other cells at any angle or position. Complex cells of this type would require more work to test for intersection, since we could no longer assume convexity, and such cells could become degenerate in a variety of ways.

To simplify the implementation, one possibility is “sampled RRM” (SRRM), which replaces the cells with finite-mass points, and replaces numerical integration with discrete summation over those points. This would take RRM’s implementation closer to that of point-based Lagrangian methods like SPH. A kernel would also be needed in order to calculate the local speed of sound used by the tracer particles. SRRM would still differ from SPH and similar Lagrangian methods in that the velocities of the mass points would be unchanged from creation until chopping. The chopping out of wavefronts would also be performed in the same way as in RRM, albeit against mass points instead of cells.

### Extension to high-performance computing architectures

The current implementation of RRM is a single-threaded event-based simulator. To run on HPC (high-performance computing) systems, which are typically made up of many thousands of computing nodes connected by a high-performance network, RRM will require parallelization.

Numerical methods based on vector and matrix operations are often amenable to intra-node parallelization via vectorization libraries such as OpenMP (Open Multi-Processing) [48]. However, event-based simulators like RRM are harder to parallelize in this way, due to the data-dependent nature of the branches in the code. So parallelization of RRM will likely take place at the inter-core level using threads, and at the inter-node level using a library such as MPI (Message Passing Interface) [49], which is widely used in the HPC community.

Previous work in the field of parallel discrete event simulators (PDES), including the seminal works of Jefferson [50] and Fujimoto [51] as well as more recent work by Barnes et al. [52], suggests how RRM could be parallelized at scale. We would divide the domain into regions, each of which would have its own event queue and run in its own thread on a dedicated core. Event queues of neighboring regions would maintain loose time synchronization, requiring a tighter synchronization handshake only at the substitution of a wavefront that spans two or more regions. The PDES literature also suggests optimizations such as the use of speculation and rollback to permit looser coupling of event queues [50].

Additional optimizations are possible in the specific case of PDES applied to RRM. The simulation domain of a physical system is simply-connected, unlike systems such as electronic circuits which may be multiply connected. This simple connectivity should make it easier to split the domain at low-activity areas to reduce the frequency of event synchronization operations. And since RRM simulates a physical system with conserved quantities, occasional non-monotonicity in event times near region boundaries may be acceptable as long as conservation is strictly maintained.

## References

[1] Walker WA. The repeated replacement method: A pure Lagrangian meshfree method for computational fluid dynamics. PLoS ONE. 2012;7(7):e39999 10.1371/journal.pone.0039999 22866175 PMC3391243

[2] Toro EF. Riemann solvers and numerical methods for fluid dynamics: a practical introduction. Springer-Verlag; 1999.

[3] Ogden R. Large deformation isotropic elasticity-on the correlation of theory and experiment for incompressible rubberlike solids In: Proceedings of the Royal Society of London A: Mathematical, Physical and Engineering Sciences. vol. 326 The Royal Society; 1972 p. 565–584.

[4] Thomas P, Lombard C. Geometric conservation law and its application to flow computations on moving grids. AIAA journal. 1979;17(10):1030–1037. 10.2514/3.61273

[5] Guillard H, Farhat C. On the significance of the geometric conservation law for flow computations on moving meshes. Computer Methods in Applied Mechanics and Engineering. 2000;190(11):1467–1482. 10.1016/S0045-7825(00)00173-0

[6] Whitehurst R. A free Lagrange method for gas dynamics. Monthly Notices of the Royal Astronomical Society. 1995;277(2):655–680. 10.1093/mnras/277.2.655

[7] Godunov SK. Reminiscences about difference schemes. Journal of Computational Physics. 1999;153(1):6–25. 10.1006/jcph.1999.6271

[8] Després B, Mazeran C. Lagrangian gas dynamics in two dimensions and Lagrangian systems. Archive for Rational Mechanics and Analysis. 2005;178(3):327–372. 10.1007/s00205-005-0375-4

[9] Maire PH, Abgrall R, Breil J, Ovadia J. A cell-centered Lagrangian scheme for two-dimensional compressible flow problems. SIAM Journal on Scientific Computing. 2007;29(4):1781–1824. 10.1137/050633019

[10] Maire PH. A high-order cell-centered Lagrangian scheme for two-dimensional compressible fluid flows on unstructured meshes. Journal of Computational Physics. 2009;228(7):2391–2425. 10.1016/j.jcp.2008.12.007

[11] Carré G, Del Pino S, Després B, Labourasse E. A cell-centered Lagrangian hydrodynamics scheme on general unstructured meshes in arbitrary dimension. Journal of Computational Physics. 2009;228(14):5160–5183. 10.1016/j.jcp.2009.04.015

[12] Kluth G, Després B. Discretization of hyperelasticity on unstructured mesh with a cell-centered Lagrangian scheme. Journal of Computational Physics. 2010;229(24):9092–9118. 10.1016/j.jcp.2010.08.024

[13] Burton D, Carney T, Morgan N, Sambasivan S, Shashkov M. A cell-centered Lagrangian Godunov-like method for solid dynamics. Computers & Fluids. 2013;83:33–47. 10.1016/j.compfluid.2012.09.008

[14] Burton DE, Morgan NR, Carney TC, Kenamond MA. Reduction of dissipation in Lagrange cell-centered hydrodynamics (CCH) through corner gradient reconstruction (CGR). Journal of Computational Physics. 2015;299:229–280. 10.1016/j.jcp.2015.06.041

[15] Vilar F, Shu CW, Maire PH. Positivity-preserving cell-centered Lagrangian schemes for multi-material compressible flows: From first-order to high-orders. Part I: The one-dimensional case. Journal of Computational Physics. 2016;312:385–415. 10.1016/j.jcp.2016.01.037

[16] Wadsley J, Veeravalli G, Couchman H. On the treatment of entropy mixing in numerical cosmology. Monthly Notices of the Royal Astronomical Society. 2008;387(1):427–438. 10.1111/j.1365-2966.2008.13260.x

[17] Berger MJ, Colella P. Local adaptive mesh refinement for shock hydrodynamics. Journal of computational Physics. 1989;82(1):64–84. 10.1016/0021-9991(89)90035-1

[18] Peraire J, Vahdati M, Morgan K, Zienkiewicz OC. Adaptive remeshing for compressible flow computations. Journal of computational physics. 1987;72(2):449–466. 10.1016/0021-9991(87)90093-3

[19] Radovitzky R, Ortiz M. Lagrangian finite element analysis of newtonian fluid flows. International Journal for Numerical Methods in Engineering. 1998;43(4):607–619. 10.1002/(SICI)1097-0207(19981030)43:4<607::AID-NME399>3.0.CO;2-N

[20] Hirt C, Amsden AA, Cook J. An arbitrary Lagrangian-Eulerian computing method for all flow speeds. Journal of computational physics. 1974;14(3):227–253. 10.1016/0021-9991(74)90051-5

[21] Barlow AJ, Maire PH, Rider WJ, Rieben RN, Shashkov MJ. Arbitrary Lagrangian—Eulerian methods for modeling high-speed compressible multimaterial flows. Journal of Computational Physics. 2016;322:603–665. 10.1016/j.jcp.2016.07.001

[22] Springel V. E pur si muove: Galilean-invariant cosmological hydrodynamical simulations on a moving mesh. Monthly Notices of the Royal Astronomical Society. 2010;401(2):791–851. 10.1111/j.1365-2966.2009.15715.x

[23] Duffell PC, MacFadyen AI. TESS: a relativistic hydrodynamics code on a moving Voronoi mesh. The Astrophysical Journal Supplement Series. 2011;197(2):15 10.1088/0067-0049/197/2/15

[24] Gaburov E, Johansen A, Levin Y. Magnetically Levitating Accretion Disks around Supermassive Black Holes. The Astrophysical Journal. 2012;758(2):103 10.1088/0004-637X/758/2/103

[25] Hopkins PF. A new class of accurate, mesh-free hydrodynamic simulation methods. Monthly Notices of the Royal Astronomical Society. 2015;450(1):53–110. 10.1093/mnras/stv195

[26] Reed WH, Hill T. Triangular mesh methods for the neutron transport equation. Los Alamos Report LA-UR-73-479. 1973;.

[27] Godunov SK. A difference method for numerical calculation of discontinuous solutions of the equations of hydrodynamics. Matematicheskii Sbornik. 1959;89(3):271–306.

[28] Barton PT, Drikakis D, Romenski E, Titarev VA. Exact and approximate solutions of Riemann problems in non-linear elasticity. Journal of Computational Physics. 2009;228(18):7046–7068. 10.1016/j.jcp.2009.06.014

[29] Lucy LB. A numerical approach to the testing of the fission hypothesis. The astronomical journal. 1977;82:1013–1024. 10.1086/112164

[30] Gingold RA, Monaghan JJ. Smoothed particle hydrodynamics: theory and application to non-spherical stars. Monthly notices of the royal astronomical society. 1977;181(3):375–389. 10.1093/mnras/181.3.375

[31] Lanson N, Vila JP. Renormalized meshfree schemes I: consistency, stability, and hybrid methods for conservation laws. SIAM Journal on Numerical Analysis. 2008;46(4):1912–1934. 10.1137/S0036142903427718

[32] Gaburov E, Nitadori K. Astrophysical weighted particle magnetohydrodynamics. Monthly Notices of the Royal Astronomical Society. 2011;414(1):129–154. 10.1111/j.1365-2966.2011.18313.x

[33] Gauger C, Leinen P, Yserentant H. The finite mass method. SIAM Journal on Numerical Analysis. 2000;37(6):1768–1799. 10.1137/S0036142999352564

[34] Klingler M, Leinen P, Yserentant H. The finite mass method on domains with boundary. SIAM Journal on Scientific Computing. 2005;26(5):1744–1759. 10.1137/S1064827502420483

[35] Klingler M, Leinen P, Yserentant H. A restart procedure for the finite mass method. SIAM Journal on Scientific Computing. 2007;30(1):117–133. 10.1137/050641235

[36] Chaikin GM. An algorithm for high-speed curve generation. Computer graphics and image processing. 1974;3(4):346–349. 10.1016/0146-664X(74)90028-8

[37] Catmull E. A subdivision algorithm for computer display of curved surfaces. DTIC Document; 1974.

[38] Titarev V, Romenski E, Toro E. Exact Riemann problem solutions and upwind fluxes for nonlinear elasticity. Preprint du Isaac Newton Institute for Mathematical Sciences NI06018-NPA 2006;.

[39] Sedov LI. Similarity and dimensional methods in mechanics. Academic Press; 1959.

[40] Taylor G. The formation of a blast wave by a very intense explosion. I. Theoretical discussion. Proceedings of the Royal Society of London Series A, Mathematical and Physical Sciences. 1950; p. 159–174. 10.1098/rspa.1950.0049

[41] Taylor G. The formation of a blast wave by a very intense explosion. II. The atomic explosion of 1945 In: Proceedings of the Royal Society of London A: Mathematical, Physical and Engineering Sciences. vol. 201 The Royal Society; 1950 p. 175–186.

[42] Alsmeyer H. Density profiles in argon and nitrogen shock waves measured by the absorption of an electron beam. Journal of Fluid Mechanics. 1976;74(03):497–513. 10.1017/S0022112076001912

[43] Heß S, Springel V. Particle hydrodynamics with tessellation techniques. Monthly Notices of the Royal Astronomical Society. 2010;406(4):2289–2311. 10.1111/j.1365-2966.2010.16892.x

[44] Frontiere N, Raskin CD, Owen JM. CRKSPH—A Conservative Reproducing Kernel Smoothed Particle Hydrodynamics Scheme. Journal of Computational Physics. 2017;332:160–209. 10.1016/j.jcp.2016.12.004

[45] Noh WF. Errors for calculations of strong shocks using an artificial viscosity and an artificial heat flux. Journal of Computational Physics. 1987;72(1):78–120. 10.1016/0021-9991(87)90074-X

[46] Laney CB. Computational gasdynamics. Cambridge university press; 1998.

[47] Woodward P, Colella P. The numerical simulation of two-dimensional fluid flow with strong shocks. Journal of computational physics. 1984;54(1):115–173. 10.1016/0021-9991(84)90142-6

[48] Dagum L, Menon R. OpenMP: an industry standard API for shared-memory programming. Computational Science & Engineering, IEEE. 1998;5(1):46–55. 10.1109/99.660313

[49] Forum MP. MPI: A Message-Passing Interface Standard; 2012.

[50] Jefferson DR. Virtual time. ACM Transactions on Programming Languages and Systems (TOPLAS). 1985;7(3):404–425. 10.1145/3916.3988

[51] Fujimoto RM. Parallel discrete event simulation. Communications of the ACM. 1990;33(10):30–53. 10.1145/84537.84545

[52] Barnes Jr PD, Carothers CD, Jefferson DR, LaPre JM. Warp speed: executing time warp on 1,966,080 cores. In: Proceedings of the 1st ACM SIGSIM Conference on Principles of Advanced Discrete Simulation. ACM; 2013. p. 327–336.

